# Stable Actinide π Complexes of a Neutral 1,4‐Diborabenzene

**DOI:** 10.1002/anie.202004501

**Published:** 2020-05-25

**Authors:** Valerie Paprocki, Peter Hrobárik, Katie L. M. Harriman, Martin S. Luff, Thomas Kupfer, Martin Kaupp, Muralee Murugesu, Holger Braunschweig

**Affiliations:** ^1^ Institut für Anorganische Chemie Julius-Maximilians-Universität Würzburg Am Hubland 97074 Würzburg Germany; ^2^ Institute for Sustainable Chemistry & Catalysis with Boron Julius-Maximilians-Universität Würzburg Am Hubland 97074 Würzburg Germany; ^3^ Institut für Chemie Theoretische Chemie/Quantenchemie, Sekr. C7 Technische Universität Berlin Straße des 17. Juni 135 10623 Berlin Germany; ^4^ Department of Inorganic Chemistry Faculty of Natural Sciences Comenius University 84215 Bratislava Slovakia; ^5^ Department of Chemistry and Biomolecular Sciences University of Ottawa 10 Marie Curie Ottawa Ontario K1N 6N5 Canada

**Keywords:** actinides, bonding, boron, heterocycles, π complexes

## Abstract

The π coordination of arene and anionic heteroarene ligands is a ubiquitous bonding motif in the organometallic chemistry of d‐block and f‐block elements. By contrast, related π interactions of neutral heteroarenes including neutral bora‐π‐aromatics are less prevalent particularly for the f‐block, due to less effective metal‐to‐ligand backbonding. In fact, π complexes with neutral heteroarene ligands are essentially unknown for the actinides. We have now overcome these limitations by exploiting the exceptionally strong π donor capabilities of a neutral 1,4‐diborabenzene. A series of remarkably robust, π‐coordinated thorium(IV) and uranium(IV) half‐sandwich complexes were synthesized by simply combining the bora‐π‐aromatic with ThCl_4_(dme)_2_ or UCl_4_, representing the first examples of actinide complexes with a neutral boracycle as sandwich‐type ligand. Experimental and computational studies showed that the strong actinide–heteroarene interactions are predominately electrostatic in nature with distinct ligand‐to‐metal π donation and without significant π/δ backbonding contributions.

## Graphical abstract

**Boracycles π‐captured**: The first actinide‐based molecules featuring π‐ligated neutral diborabenzene (dbb) as sandwich‐type ligand are reported. Experimental and theoretical studies suggest that the unique strength of the actinide–dbb bond is mainly electrostatic in nature with distinct covalent ligand‐to‐metal orbital contributions.

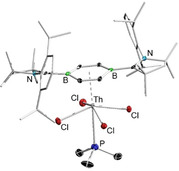

## Introduction

**Figure 1:**
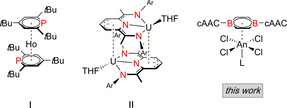
Stable f‐block element π complexes with (formally) neutral heteroarene ligands (Ar=mesityl; An=Th, U).

The π‐type complexation of aromatic carbocycles by d‐block and f‐block metal centers takes a unique position in the history of organometallic chemistry with landmark moments such as the discoveries of ferrocene,^1^ bis(benzene)chromium^2^ and uranocene.^3^ In fact, this concept was one of the first that has been successfully transferred from transition metal to actinide chemistry,3b, 4 thus such species have always been of high value for studying f‐element‐ligand bonding and determining critical parameters such as the extent of 5f‐orbital participation and metal‐ligand covalency. Nowadays, most prototypic aromatic carbocycles have been incorporated as unsupported sandwich‐type ligands into numerous actinide π complexes^5^ including anionic C_4_–C_8_ 6 and neutral C_6_ rings,^7^ as well as anionic fused aromatics such as naphthalene^8^ or pentalene.^9^ When it comes to related heteroarene complexes, the diversity becomes significantly smaller, and a strong imbalance in favor of the d‐block transition metals is encountered. Thus, π‐ligated heteroarene complexes of the d‐elements have been realized for a large number of aromatic heterocycles all across the periodic table including B‐based systems (BNC_2_, B_2_N_2_, BC_4_, BNC_3_, BC_5_, B_2_C_4_, BNC_4_, BC_6_)^10^ and benzene analogs EC_5_ (E=B‐Ga, Si‐Sn, N‐Sb),^11^ to name only a few. For the f‐elements, π complexation of anionic BNC_3_,^12^ BC_5_,^13^ AlC_5_,^14^ NC_4_,5h, 7i, 15 N_2_C_3_,^16^ C_2_P_2_,6a PC_4_,^17^ P_3_C_2_,^18^ PN_2_C_2_,^19^ P_4_/As_4_,^20^ and P_5_ 21 has been verified for selected lanthanide and actinide molecules. By contrast, f‐block complexes bearing neutral heteroarenes as sandwich‐type ligands are exceedingly rare, and limited to [(*t*Bu_3_‐C_5_H_2_P)_2_Ho] (**I**).^22^ Pyridine(diamine) uranium species of the type **II** formally also contain a neutral nitrogen heterocycle, however, π coordination to uranium involves reduced anionic pyridine rings (Figure 1).^23^ We note that π complexation of boracycles is still unknown for the actinides in general.

At this point, we wondered what requirements had to be met by the actinide metal center and a neutral heteroaromatic ligand to create more stable π interactions. In general, the strength of metal‐arene π bonding is dictated by two factors: (i) Electrostatics, which explains the preference of “hard” actinide cations for π complexation of “hard” anionic (hetero)arene ligands. (ii) Metal‐to‐ligand backdonation, which is the dominant part of bonding interactions in π complexes of neutral (hetero)arenes.^24^ We reasoned that the electrostatic term is maximized by employing high‐oxidation‐state metal precursors (Th^IV^, U^IV–VI^), which, at the same time, will limit ligand reduction processes, thus allowing the generation of species with truly neutral heteroaromatic ligands. This, however, will significantly lower the backdonation capabilities of the actinide metal center, thus electron‐rich heteroarenes with very strong π donor strengths will be required as antidote. Recent studies in our group have highlighted the exceptional π donor strength of the bora‐π‐aromatic 1,4‐bis(cAAC)_2_‐1,4‐diborabenzene [**1**; dbb; cAAC=cyclic (alkyl)(amino)carbene]^25^ in remarkably stable Group 6 half‐sandwich complexes [(dbb)M(CO)_3_] (M=Cr, Mo, W).10s We were thus confident that the dbb ligand might be a suitable choice for generating the first stable actinide π complexes with neutral heteroarene ligands.

## Results and Discussion

**Scheme 1:**
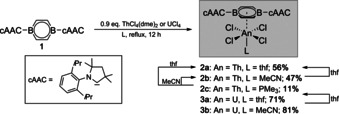
Reactivity of dbb **1** with ThCl_4_(dme)_2_ and UCl_4_ to afford actinide half‐sandwich complexes **2** and **3**.

When ThCl_4_(dme)_2_ and UCl_4_ were allowed to react with 1.1 equivalents of the neutral bora‐π‐aromatic **1** in a donor solvent (thf, MeCN) at refluxing conditions (12 h), either purple suspensions (Th) or deep‐red solutions (U) formed, from which π complexes [(dbb)(L)AnCl_4_] (**2 a**: An=Th, L=thf; **2 b**: An=Th, L=MeCN; **3 a**: An=U, L=thf; **3 b**: An=U, L=MeCN) were isolated as red solids in moderate to good yields (Scheme 1). Compounds **2 a**/**b** and **3 a**/**b** are thermally robust, even in the presence of an excess of the respective donor solvent, which strongly contrasts with the labile π coordination of benzene and its methylated analogs in related species such as [(*η* ^6^‐C_6_H_n_Me_6−*n*_)UX_3_] (X=BH_4_, AlCl_4_),7a, 7d and [(*η* ^6^‐C_6_Me_6_)_2_U_2_Cl_7_][AlCl_4_].7b However, when dissolved in thf, the acetonitrile ligand of **2 b** and **3 b** is readily displaced quantitatively to afford thf complexes **2 a** and **3 a**. No changes were observed upon dissolving **2 a** and **3 a** in MeCN. This reactivity is not surprising given the better σ donor properties of thf, and the oxophilicity of the actinides. The fact that ligand displacement reactions preferably occur at the Lewis base site of [(dbb)(L)AnCl_4_] without affecting the π coordination of the dbb ligand is remarkable, and clearly emphasizes the unique strength of these π interactions.

By contrast, complexes **2 a**/**b** and **3 a**/**b** proved highly sensitive under redox conditions. In our hands, chemical oxidation or reduction consistently led to decomposition of **2 a**/**b** and **3 a**/**b** to afford free dbb **1** and unknown actinide species. It should be noted that **2 a**/**b** and **3 a**/**b** were also formed when the reactions were carried out in chlorinated (CH_2_Cl_2_) or aromatic solvents (benzene/toluene) in the presence of 1 equivalent of thf/MeCN, although yields were lower in these cases. In the absence of donor solvents, however, no reaction occurred for UCl_4_ (presumably because of its low solubility), and ThCl_4_(dme)_2_ is partly converted to the dme‐bridged dimer [{(dbb)ThCl_4_}_2_‐*κ*‐dme] (**4**) (optimized conditions: fluorobenzene, Δ*T*, 20 h, 10 % isolated yield; Figure S23). Thus, our initial experiments indicated that actinide π complexes of neutral diborabenzene **1** are readily accessible simply by combining the ligand with standard actinide reactants. We note that the simplicity of this approach is very uncommon in condensed phase keeping in mind that the π complexation process usually requires pre‐activation of the metal center under ligand abstracting conditions such as reduction, oxidation, photolysis, or halide abstraction.

We next turned our attention to the electronic structure of the actinide metal centers of **2 a**/**b** and **3 a**/**b**. Formally, oxidation states of +IV are required to exclude the occurrence of ligand reduction processes upon dbb coordination, and to ascertain the neutral nature of the dbb π ligand. For **2 a**/**b**, their chemical composition and solution NMR spectra in the normal diamagnetic range strongly indicate an oxidation state of +IV for the thorium centers, even though a coupled biradical character due to non‐innocence of the dbb ligand cannot be ruled out completely. The ^1^H NMR spectra of **2 a**/**b** confirm the presence of a 1:1 ratio of coordinated dbb and Lewis base with their expected signal patterns. Noteworthy are the chemical shifts for the aromatic dbb ring protons (**2 a**: *δ*_H_=7.18; **2 b**: *δ*_H_=7.78), which almost remain unaltered from that of the free ligand **1** (*δ*_H_=7.31). Similarly, the ^11^B NMR resonances of the boron nuclei (**2 a**: *δ*_B_=27.5; **2 b**: *δ*_B_=27.8) are only slightly shifted to higher frequencies upon complexation (**1**: *δ*_B_=24.8).^25^ By contrast, the related Group 6 half‐sandwich complexes [(dbb)M(CO)_3_] (M=Cr, Mo, W) exhibited a significant high‐field shift of both the ^1^H (*δ*_H_=4.74–4.97) and ^11^B NMR (*δ*_B_=6.0–7.0) resonances of the diborabenzene ligand.10s This behavior was interpreted in terms of strong metal‐to‐ligand backbonding contributions from the electron‐rich Group 6 metal centers to the empty dbb ligand orbitals, thus creating highly covalent bonding interactions. Consequently, the present findings indicate a fundamentally different bonding picture for **2 a**/**b** with larger electrostatic and rather small metal‐to‐ligand backbonding contributions, which is in line with the higher oxidation state of Th^IV^ and its lack of f electrons.

**Figure 2:**
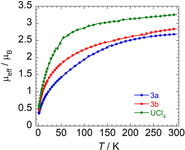
Temperature‐dependent SQUID magnetization data (at B=1000 Oe) of U^IV^ complexes **3 a**, **3 b** and UCl_4_, as a function of the effective magnetic moment (*μ*_eff_) versus the temperature (*T*).

For **3 a**/**b**, magnetic susceptibility measurements also account for an oxidation state of +IV of the uranium centers, thus verifying the presence of neutral dbb π ligands in **3 a**/**b** as well. In solution, **3 a**/**b** show paramagnetic behavior at room temperature with paramagnetically shifted and broadened NMR resonances (**3 a**: *δ*_B_=−46.0; **3 b**: *δ*_B_=−70.0), and effective magnetic moments consistent with the presence of two unpaired electrons (*cf*. **3 a**: *μ*_eff_=2.61 *μ*_B_; Evans NMR method, CD_2_Cl_2_).^26^ SQUID magnetization data of **3 a**/**b** in the solid state are also consistent with 5f^2^d^0^ (^3^H_4_) electron configurations (Figures 2, S24, S25). Accordingly, *μ*_eff_ gradually decreases from 2.685 *μ*_B_ (**3 a**) and 2.840 *μ*_B_ (**3 b**) at 300 K, followed by a rapid decrease below 50 K to values of 0.371 *μ*_B_ (**3 a**) and 0.488 *μ*_B_ (**3 b**) at 1.8 K, resulting in curvatures reminiscent of U^IV^ complexes (see Supporting Information for a more detailed discussion of SQUID data).

**Figure 3:**
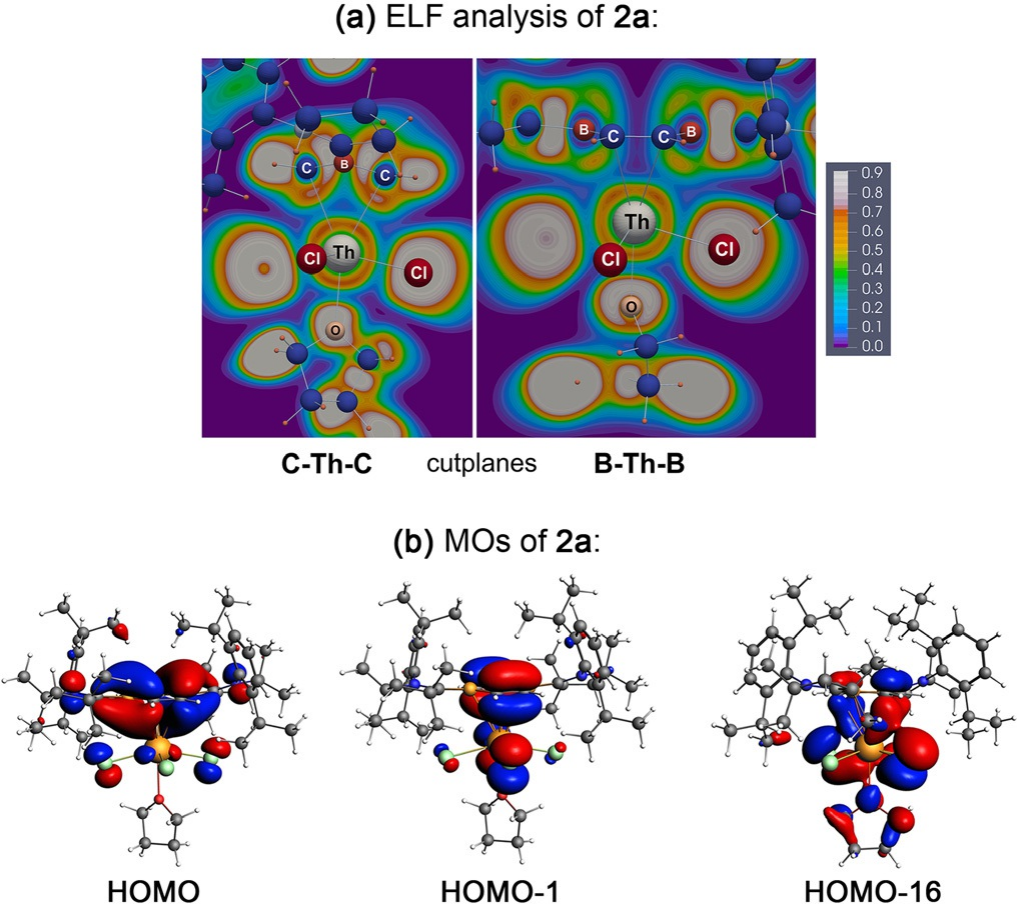
a) Cut‐plane plots from the ELF analysis of **2 a**. Gray‐white regions represent areas near ELF maxima (bonding attractors). b) Frontier molecular orbital representations of **2 a** relevant to actinide‐heteroarene bonding: HOMO, HOMO−1, and HOMO−16.

The exact nature of the An–dbb π interaction in complexes **2 a**/**b** and **3 a**/**b** was assessed by DFT calculations. To this end, we studied the electronic structures of **1**, **2 a**, **3 a**, the hypothetical benzene analogues [(*η* ^6^‐C_6_H_6_)(thf)AnCl_4_] (An=Th, U), and some literature‐known [(*η* ^6^‐C_6_H_*n*_Me_6−*n*_)UX_3_] (X=BH_4_, AlCl_4_) species, applying 5f^0^d^0^, 5f^2^d^0^ and 5f^3^d^0^ electron configurations for the Th^IV^, U^IV^, and U^III^ centers, respectively. The computed structural and spectroscopic parameters of **2 a** and **3 a** agree very well with experimentally determined values (Supporting Information). The calculations suggest that the An–dbb interactions in **2 a** and **3 a** should be viewed as largely electrostatic in nature with small, but distinct orbital contributions, which coincides with only marginal changes in NMR shifts after complexation of dbb by Th^IV^. Thus, delocalization indices (QTAIM DIs), which serve as a measure of the bond covalency for a given pair of atoms,^27^ show rather small values for the An−C bonds (**2 a**: 0.174; **3 a**: 0.200) in comparison to regular covalent An−C bonds.27b However, An−C bond covalency of **2 a** and **3 a** notably exceed those calculated for [(*η* ^6^‐C_6_H_6_)U(AlCl_4_)_3_] (av. DIs=0.141) or hypothetical [(*η* ^6^‐C_6_H_6_)(thf)AnCl_4_] [av. DIs=0.083 (Th), 0.094 (U)] and approach, for instance, An−C bond covalencies computed for [Cp_4_An] [av. DIs=0.190 (Th), 0.219 (U)] with negatively charged Cp ligands (Supporting Information). Albeit weak in nature, covalent An–B contributions to the An–dbb interaction cannot be entirely neglected [av. DIs=0.050 (**2 a**), 0.055 (**3 a**)], as further illustrated by the presence of Th−B bonding attractors in **2 a** (ELF analysis; Figure 3 a). The predominance of the electrostatic term also becomes evident in a significant decrease of the partial ring charges upon π complexation of dbb from −0.576 (**1**) to −0.289 (**2 a**) and −0.260 (**3 a**), which is consistent with strong ligand‐to‐metal electron donation.

**Figure 4:**
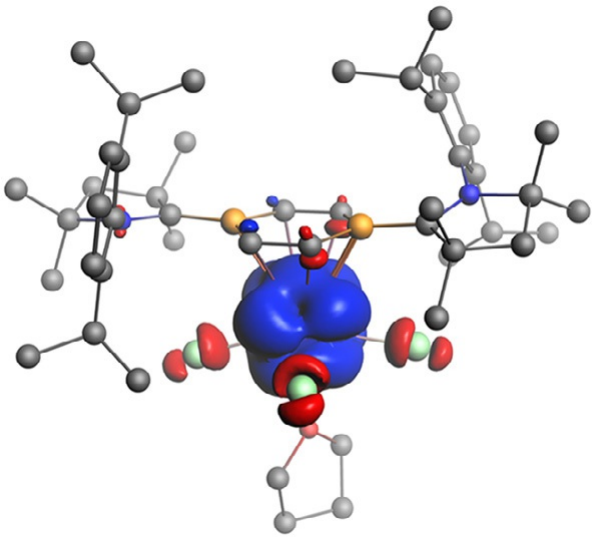
Spin‐density distribution in the triplet ground‐state of **3 a** (isosurface plot ±0.001 a.u.; blue surface indicates positive spin density and red indicates negative spin density). Hydrogen atoms are omitted for clarity.

Inspection of the frontier molecular orbitals (MOs) of **2 a** and **3 a** helped to identify covalent contributions to the An–dbb bonding interaction. For **2 a**, only three relevant MOs (HOMO, HOMO−1, HOMO−16; Figures 3 b, S26) were located, all reflecting the significance of ligand‐to‐metal π donation. Hence, thorium d/f orbital participation is rather low in these MOs [HOMO: 4 % Th(d), HOMO−1: 3 % Th(f), HOMO−16: 11 % Th(d)], and their shape resembles that of the frontier molecular orbitals HOMO and HOMO−1 of free dbb **1**.^25^ While HOMO of **2 a** illustrates the π donor interaction of the delocalized aromatic π system of **1** (HOMO) to thorium's vacant 6d orbitals, HOMO−1 and HOMO−16 are reflective of ligand‐to‐metal π bonding emanating from C=C‐centered ligand π orbitals (HOMO−1) to empty 5f and 6d orbitals of thorium. It should be emphasized here that MOs associated with metal‐to‐ligand π/δ backbonding could not be located by our calculations. Similar interactions were also derived for uranium complex **3 a** (Figure S27), while the presence of two f electrons in principle allows for metal‐to‐ligand backbonding interactions. Spin‐density calculations, however, have shown that the two unpaired f electrons predominately reside at the U^IV^ center with small negative spin densities at chlorine atoms (Figure 4), making such metal‐to‐ligand π/δ backbonding contributions rather weak in nature.

The unique strength of the An–heteroarene interaction was also evaluated by energy decomposition analysis (EDA),^28^ which showed that the total bonding energy Δ*E*_int_ of the diborabenzene ligand in **2 a** (−276.9 kJ mol^−1^) and **3 a** (−283.2 kJ mol^−1^) is roughly three times that of the benzene ligand in hypothetical [(*η* ^6^‐C_6_H_6_)(thf)AnCl_4_] [−94.4 kJ mol^−1^ (Th); −105.2 kJ mol^−1^ (U)] or that of the thf ligand of **2 a**/**3 a** *trans* to the dbb ligand [−85.8 kJ mol^−1^ (**2 a**); −87.8 kJ mol^−1^ (**3 a**)], and still significantly larger than that of benzene and hexamethylbenzene in literature‐known examples [(*η* ^6^‐C_6_H_6_)U(AlCl_4_)_3_] (−176.9 kJ mol^−1^) and [(*η* ^6^‐C_6_Me_6_)U(BH_4_)_3_] (−199.4 kJ mol^−1^), respectively. In contrast, the thf ligand is more tightly bound than the arene ligand in [(*η* ^6^‐C_6_H_6_)(thf)AnCl_4_] [Δ*E*_int_=−129.2 kJ mol^−1^ (Th), −159.8 kJ mol^−1^ (U)], which makes such species experimentally inaccessible. Thus, the donor capabilities of the diborabenzene ligand are superior to those of benzene, its methylated analogs (mesitylene, C_6_Me_6_), and thf, which explains the high stability of complexes **2 a** and **3 a** even in coordinating solvents.

**Figure 5:**
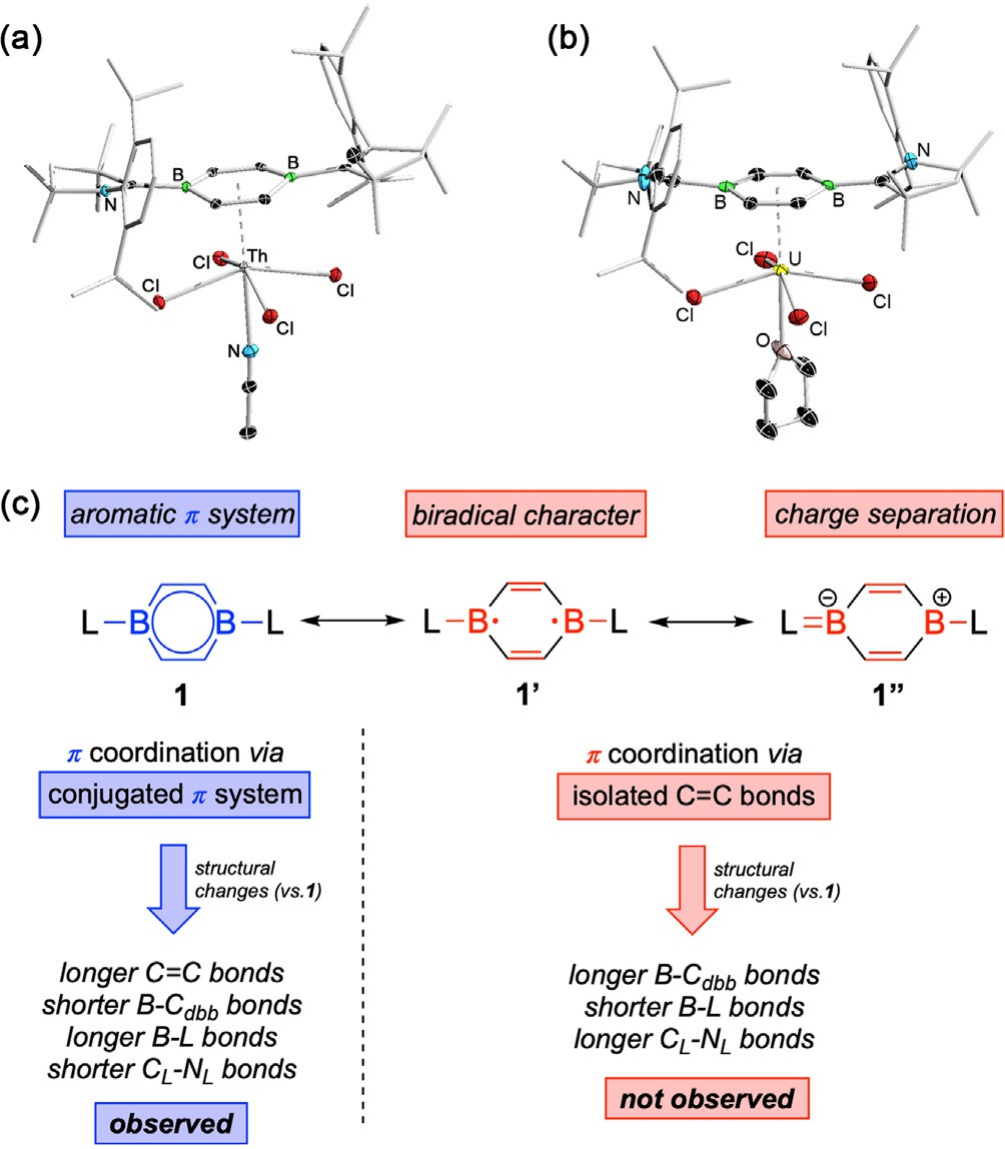
a,b) Solid‐state structures of **2 b** and **3 a**. Hydrogen atoms and some of the ellipsoids of the cAAC ligand have been omitted for clarity. c) Possible resonance structures (conjugated **1**, biradical **1′**, charge separated **1′′**) and resulting π coordination mode of dbb **1**.

The isolation of crystalline **2 a**/**b** and **3 a**/**b** allowed us to elucidate their solid‐state structures by X‐ray diffraction analyses (Figures 5 a/b, S18, S22). All complexes exhibit pseudo‐octahedral geometries with four chloride ligands in equatorial positions, and one molecule each of Lewis base L (thf, MeCN) and dbb mutually *trans* in axial positions. Unexpectedly, the dbb heteroarene is not perfectly planar, instead complexation results in minor deviations of the dbb ligand from planarity in all cases, that is, the two boron atoms are slightly bent out of the ring plane (6.1 to 8.8°) away from the metal center. Thus, the hapticity of the An–dbb π coordination seems to be best described as *η* ^4^ with close An–C_dbb_ contacts. However, theoretical evidence of weak covalent An–B interactions suggests that the bonding picture is not that simple and that *η* ^6^‐type contributions have to be considered as well. Hence, the true bonding situation most likely lies within the *η* ^4^‐*η* ^6^‐continuum, but definitely on the *η* ^4^‐side.

Notwithstanding its hapticity, theoretical and experimental considerations clearly show that the diborabenzene ligand is bound to the actinide centers of **2 a**/**b** and **3 a**/**b** via its fully conjugated π system, and not via interaction of the actinide metal centers with two isolated C=C double bonds of the heteroarene, as might be reasoned from strong *η* ^4^‐contributions. First of all, our computations emphasize the significance of ligand‐type orbitals for An–dbb bonding, which mainly involve HOMO and HOMO−1 of free dbb **1**, orbitals of π symmetry spanning the whole B_2_C_4_ heterocyclic backbone (resonance structure **1**, Figure 5 c). More importantly, the type of π coordination active in molecules **2 a**/**b** and **3 a**/**b** is expected to directly affect their spectroscopic and structural properties. Hence, interaction of the actinide centers with two isolated C=C double bonds would require the unfavorable breakup of aromatic π conjugation within dbb, resulting in unfavorable biradical or charge separated resonance structures **1′** and **1′′** (Figure 5 c). In our hands, the presence of such resonance structures can be excluded for **2 a**/**b** and **3 a**/**b**. While any biradical character (**1′**) can be ruled out on the basis of EPR spectroscopic studies, charge separation (**1′′**) appears very unlikely when closely inspecting the solid‐state structures of **2 a**/**b** and **3 a**/**b**. Thus, π coordination of dbb via resonance structures **1′** and **1′′** most likely causes significant elongation of the endocyclic B−C_dbb_ of the dbb ligand, while, as a consequence, exocyclic B−C_cAAC_ and C_cAAC_−N_cAAC_ bonds will become shorter and longer, respectively. For **2 a**/**b** and **3 a**/**b** however, the opposite is true, and B−C_dbb_ (1.507(7)–1.526(3) Å) and C_cAAC_−N_cAAC_ distances (1.313(6)–1.322(5) Å) are smaller than in **1**, while C_dbb_=C_dbb_ (1.395(4)–1.403(6) Å) and B−C_cAAC_ (1.584(6)–1.597(5) Å) distances become larger (*cf*. **1**: B−C_dbb_ 1.522(3)–1.540(3) Å; C_dbb_=C_dbb_ 1.372(3), 1.378(3) Å; B−C_cAAC_ 1.554(3), 1.563(3) Å; C_cAAC_−N_cAAC_ 1.335(3), 1.346(3) Å).^25^ Consequently, X‐ray diffraction data clearly support our theoretical findings that An–dbb π coordination involves the whole aromatic B_2_C_4_ framework.

IR spectroscopic studies on **1**, **2 a**/**b** and **3 a**/**b** in the solid state also support this π bonding picture (Figures S12–S17). Here, IR bands associated with the endocyclic C=C bonds are shifted to lower energies upon complexation of dbb, that is, from 1412–1472 cm^−1^ in **1** to 1365–1423 cm^−1^ in **2 a**/**b** and **3 a**/**b**. At the same time, the strong IR absorption of the C_cAAC_−N_cAAC_ bond is shifted to higher wavenumbers (*cf*. **1**: 1423 cm^−1^; **2 a**/**b**, **3 a**/**b**: 1454–1458 cm^−1^), which is consistent with stronger C_cAAC_−N_cAAC_ bonds in π complexes **2 a**/**b** and **3 a**/**b** (assignment of IR bands supported by by frequencies calculations).

An−C bond lengths were determined to be in the range of 2.831(2) to 2.948(4) Å (**2 a**: Th–dbb_cent_ 2.586 Å; **2 b**: Th–dbb_cent_ 2.556 Å; **3 a**: U–dbb_cent_ 2.585 Å; **3 b**: U–dbb_cent_ 2.490 Å). We note that these contacts are quite short, which illustrates the strong actinide–heteroarene interaction in **2 a**/**b** and **3 a**/**b**. For **2 a**/**b**, a CSD search on Th complexes featuring neutral π arene ligands provided reasonably longer Th–C_cent_ distances (2.706–2.950 Å).7g, 7h, 7l, 7m The U–C distances of **3 a**/**b**, however, strongly resemble those in [(*η* ^6^‐C_6_Me_6_)UX_3_] (X=BH_4_, AlCl_4_; av. U–C 2.92 Å),7a, 7d and [(*η* ^6^‐C_6_Me_6_)_2_U_2_Cl_7_][AlCl_4_] (av. U–C 2.92 Å).7b Long An–B separation distances (**2 a**: 3.049(3) Å; **2 b**: 3.036(4) Å; **3 a**: 3.048(5) Å; **3 b**: 2.979(3) Å) are in agreement with theory and rather weak An−B bonding interactions. Overall, experimental and theoretical data suggest that neutral dbb is tightly bound to Th^IV^ and U^IV^ in a *η* ^4^‐type coordination mode via π interactions involving the whole aromatic π system (mediated primarily by electrostatics in combination with distinct covalent bonding contributions; ligand‐to‐metal donation; no notable π/δ backbonding).

Finally, we set out to overcome the well‐known tendency of actinide ions to preferably bind “hard” donor ligands and tried to incorporate the “soft” Lewis base PMe_3_ in dbb complexes of the type [(dbb)(L)AnCl_4_]. Thus, the reactions of ThCl_4_(dme)_2_ and UCl_4_ with 1.1 equivalent of dbb **1** in the presence of PMe_3_ in benzene under refluxing conditions resulted in the generation of PMe_3_‐substituted species [(dbb)(PMe_3_)AnCl_4_] in solution. Due to labile An−PMe_3_ bonds, however, only [(dbb)(PMe_3_)ThCl_4_] (**2 c**) exhibited sufficient stability to allow isolation (in low yields of 11 %) (Scheme 1, Supporting Information), while its uranium analog eluded isolation and could only be observed spectroscopically.

**Figure 6:**
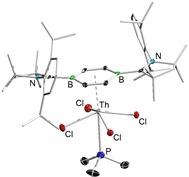
Solid‐state structure of **2 c**. Hydrogen atoms and some of the ellipsoids of the cAAC ligand have been omitted for clarity.

Red crystalline **2 c** represents the most sensitive and least stable species in the [(dbb)(L)AnCl_4_] series, readily reacting in polar/coordinating solvents, and decomposing under vacuum conditions, making its purification extremely difficult. Nevertheless, its identity was clearly verified by NMR spectroscopy and X‐ray diffraction studies (Figure 6). In solution, diamagnetic **2 c** shows a ^11^B NMR resonance at *δ*_B_=27.7 for the π ligated diborabenzene ligand (*cf*. **2 a**: *δ*_B_=27.5; **2 b**: *δ*_B_=27.8). The Th^IV^–PMe_3_ interaction of **2 c** is characterized by a ^31^P NMR signal with a chemical shift of *δ*_P_=−30.6 in solution, and by a Th−P bond distance of 3.053(1) Å in its solid‐state structure. Other structural parameters are roughly the same as those of compounds **2 a** and **2 b**. We were surprised to find that dative Th‐P interactions are still rare, and only one paper has been published reporting related stable Th−P dative bonding interactions involving non‐chelating tertiary phosphine ligands, that is, [(BH_4_)_4_Th(PR_3_)_2_] (R=Me, Et).^29^ In addition, only a few species containing the bidentate 1,2‐bis(dimethylphosphino)ethane ligand (dmpe) are known that are suitable for comparison.^30^ Here, ^31^P NMR chemical shifts range from *δ*_P_=−33.3 to −4.5 (cf. [(BH_4_)_4_Th(PMe_3_)_2_]: *δ*_P_=−22.2), and Th–P bond lengths range from 3.096(3) Å in [(BH_4_)_4_Th(PEt_3_)_2_] to 3.237(2) Å in [Cp_2_(CH_2_Ph)_4_Th(dmpe)_2_]. Nevertheless, the Th–P interaction of **2 c** must still be considered rather weak, and the PMe_3_ ligand is prone to dissociation in the presence of “hard” Lewis bases. When dissolved in either thf or MeCN, the “soft” PMe_3_ ligand is replaced instantaneously, and **2 c** converts quantitatively into its analogs **2 a** and **2 b**, respectively, which is consistent with the preferred coordination of “hard” donor ligands to thorium.

## Conclusion

In summary, we have succeeded in the realization of the first actinide‐based molecules with an aromatic boracycle as sandwich‐type π ligand, [(dbb)(L)AnCl_4_]. Complexes **2 a**–**c** and **3 a**/**b** are remarkably stable even in the presence of coordinating solvents, which contrasts with the labile π coordination often observed for related species with unsupported benzene ligands. Thus, ligand displacement reactions proceeded at the Lewis basic site in *trans*‐position to the dbb ligand without affecting actinide–heteroarene bonding. A combination of experimental and theoretical techniques was used to verify the neutral nature of the diborabenzene ligand and its π‐type coordination to the Th^IV^ and U^IV^ metal centers. The unique strength of the actinide‐heteroarene interaction is closely related to the outstanding π donor capabilities of the aromatic dbb heterocycle, thus enabling (i) strong electrostatic interactions with the electron‐poor actinide centers, and (ii) distinct covalent orbital interactions primarily via ligand‐to‐metal electron donation and without notable backbonding contributions.

## Conflict of interest

The authors declare no conflict of interest.

## Supporting information

As a service to our authors and readers, this journal provides supporting information supplied by the authors. Such materials are peer reviewed and may be re‐organized for online delivery, but are not copy‐edited or typeset. Technical support issues arising from supporting information (other than missing files) should be addressed to the authors.

Supplementary
