# Supplementary material for: Stable Actinide π Complexes of a Neutral 1,4‐Diborabenzene
Source: Angew Chem Int Ed Engl. 2020 May 25;59(31):13109–15. doi: 10.1002/anie.202004501 (PMC7496575; doi:10.1002/anie.202004501)
Supplement: Supplementary file 1 — Supplementary [file ANIE-59-13109-s001.pdf]

Angewandte Chemie
Eine Zeitschrift der Gesellschaft Deutscher Chemiker

Supporting Information

Stable Actinide π Complexes of a Neutral 1,4-Diborabenzene

Valerie Paprocki, Peter Hrobárik, Katie L. M. Harriman, Martin S. Luff, Thomas Kupfer, Martin Kaupp, Muralee Murugesu, and Holger Braunschweig**

anie_202004501_sm_miscellaneous_information.pdf

S1 Synthetic details and characterization of compounds

General experimental considerations: All manipulations were performed either under an atmosphere of dry argon, or in vacuo using standard Schlenk line or glovebox techniques. Deuterated solvents were dried over molecular sieves and degassed by three freeze-pump-thaw cycles prior to use. All other solvents were distilled and degassed from appropriate drying agents. Solvents (both deuterated and non-deuterated) were stored under argon over activated 4 Å molecular sieves. All glassware was oven-dried prior to use. Celite was dried under dynamic vacuum by heating to 150 °C for 24 h. Diborabenzene **1** (dbb),[1] $ThCl_4(dme)_2$,[2] and UCl_4[3] were prepared according to literature methods. NMR spectra were acquired on a Bruker Avance 500 NMR spectrometer (^1H: 500.1 MHz, ^{11}B: 160.5 MHz, ^{13}C: 125.8 MHz, ^{31}P: 202.4 MHz), or on a Bruker Avance 400 NMR spectrometer (^1H: 400.1 MHz, ^{11}B: 128.4 MHz, ^{31}P: 161.9 MHz). Chemical shifts (δ) are given in ppm and internally referenced to the carbon nuclei ($^{13}C\{^1H\}$) or residual protons (^1H) of the solvent. NMR spectra were referenced to $SiMe_4$ (^1H, ^{13}C), $BF_3 \cdot OEt_2$ (^{11}B), and 85% H_3PO_4 (^{31}P) as external standards. ^1H and $^{13}C\{^1H\}$ NMR signals were assigned with assistance of DEPT-135 and HSQC NMR experiments. Resonances are given as singlet (s), doublet (d), triplet (t), septet (sept) or multiplet (m). Microanalyses (C, H, N) were performed on an Elementar vario MICRO cube elemental analyser. IR spectra were acquired in the argon atmosphere of a glovebox on a Bruker Alpha spectrometer equipped with an ATR module in the solid state. Signals are given in cm^{-1}, and their intensities are denoted as weak (w), medium (m), and strong (s).

Synthesis and characterization of [(dbb)(thf)$ThCl_4$] 2a

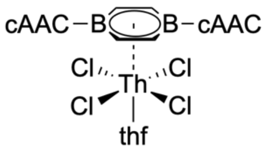

dbb (**1**) (118 mg, 184 μmol), and $ThCl_4(dme)_2$ (100 mg, 180 μmol) were suspended in thf (15 mL), and heated at reflux over a period of 12 h. The resulting purple suspension was subsequently reduced to about 2 mL in volume and transferred onto a pad of Celite. After washing with pentane (12 mL), the residue was extracted into benzene. All volatiles were evaporated from the benzene solution in vacuo to afford **2a** as a red solid (109 mg, 100 μmol, 56%). Crystals suitable for X-ray diffraction were obtained by slow evaporation of saturated CH_2Cl_2 solutions of **2a** containing few drops of benzene at −30°C. **Note:** All actinide-diborabenzene compounds extensively stuck to the glassware, for which reason highest yields of powdered samples were obtained by lyophilization from benzene.

^{1}H NMR (500.1 MHz, CD_2Cl_2): δ = 0.94 [d, 12H, $C^{Dipp}(CH(CH_3)_2)$, $^3J_{HH}$ = 4.42 Hz], 1.23 [d, 12H, $C^{Dipp}(CH(CH_3)_2)$, $^3J_{HH}$ = 6.63 Hz], 1.44 [s, 12H, $NC(CH_3)_2$], 1.83 [m, 4H, $O^{thf}C(CH_2)$], 1.92 [s, 12H, $C^{caac}C(CH_3)_2$], 2.16 [s, 4H, CH_2], 2.98 [m, 4H, $C^{Dipp}CH(CH_3)_2$], 4.33 [m, 4H, $O^{thf}(CH_2)$], 7.18 [m, 4H, $B_2(CH)_4$], 7.30 [d, 4H, *m*-$C^{Dipp}H$, $^3J_{HH}$ = 7.74 Hz], 7.48 [t, 2H, *p*-$C^{Dipp}H$, $^3J_{HH}$ = 7.74 Hz].

^{13}C NMR (125.8 Hz, CD_2Cl_2): δ = 25.5 [$C^{Dipp}(CH(CH_3)_2)$], 25.7 [$O^{thf}C(CH_2)$], 27.5 [$C^{Dipp}(CH(CH_3)_2)$], 29.3 [$NC(CH_3)_2$], 29.7 [$C^{Dipp}(CH(CH_3)_2)$], 32.6 [$C^{Carben}C(CH_3)_2$], 54.1 [$C^{caac}(CH_3)_2$], 54.4 [CH_2], 74.0 [$O^{thf}(CH_2)$], 78.5 [$NC(CH_3)_2$], 126.3 [*m*-C^{Dipp}], 130.2 [*p*-C^{Dipp}], 136.1 [*i*-C^{Dipp}], 146.1 [*o*-C^{Dipp}], 150.6 [$B_2(CH)_4$].

^{11}B NMR (160.5 MHz, CD_2Cl_2): δ = 27.5.

IR (solid): 1365, 1388, 1423 (m, ν_{dbb} C=C), 1456 (s, ν_{cAAC} C-N), 2868, 2930, 2964 (m, ν_{dbb} C-H).

Elemental analysis calculated for $C_{48}H_{74}B_2Cl_4ON_2Th\cdot 2thf$: C 54.47 H 7.35 N 2.27; Found: C 54.50 H 7.40 N 2.57.

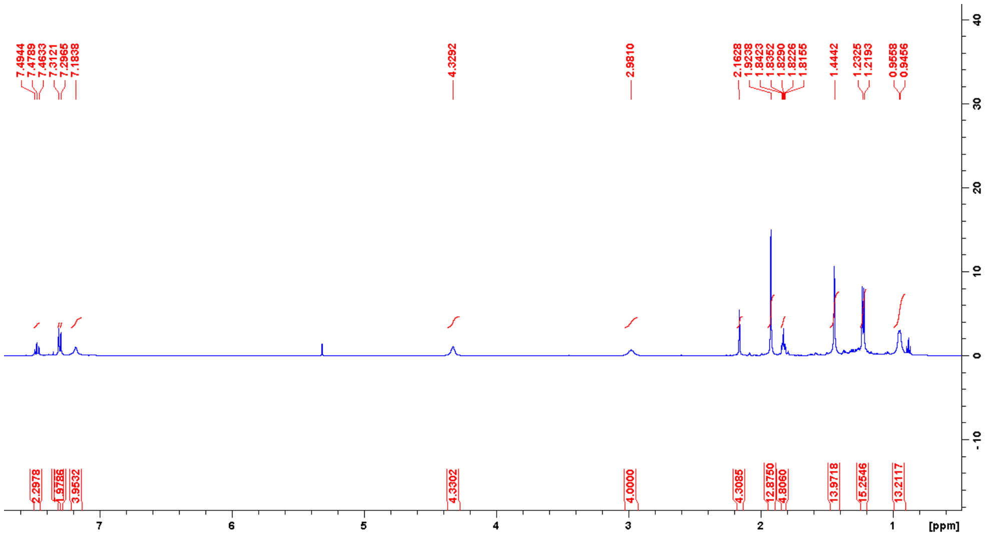

Figure S1. ^{1}H NMR spectrum of **2a** in CD_2Cl_2.

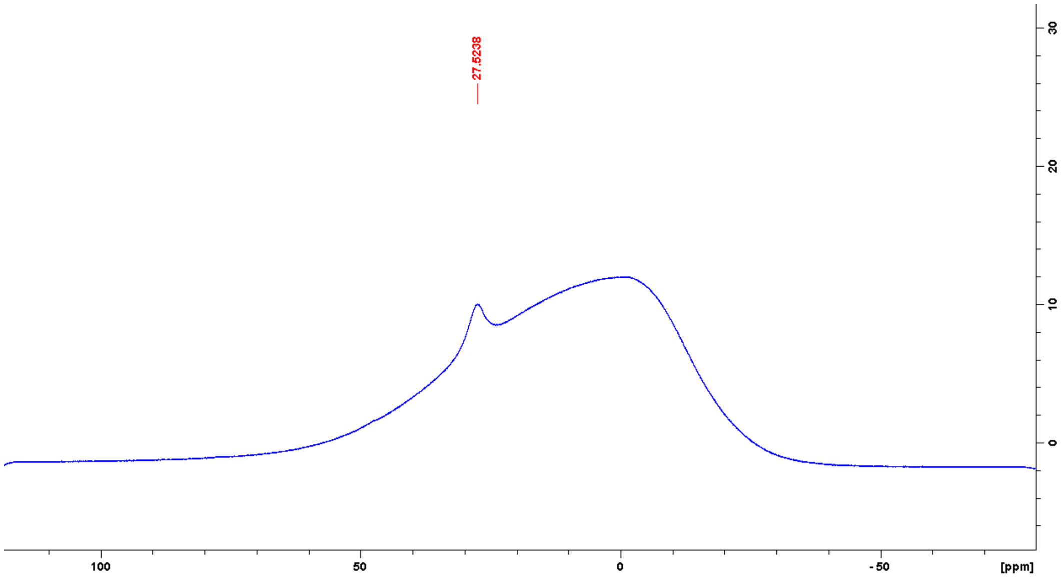

Figure S2. ^{11}B NMR spectrum of **2a** in CD_2Cl_2.

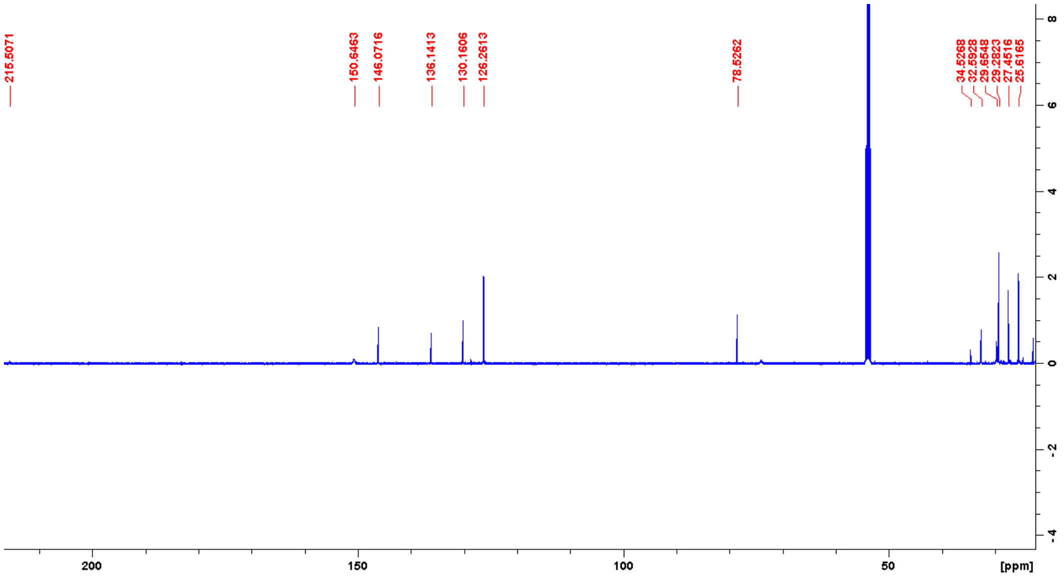

Figure S3. ^{13}C NMR spectrum of **2a** in CD_2Cl_2.

Synthesis and characterization of [(dbb)(MeCN)$ThCl_4$] 2b

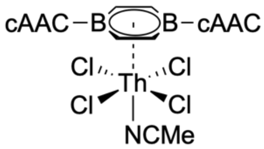

dbb (**1**) (64.0 mg, 99.3 μmol), and $ThCl_4(dme)_2$ (50.0 mg, 90.0 μmol) were suspended in MeCN (10 mL), and heated at reflux over a period of 12 h. The resulting purple suspension was subsequently reduced to about 2 mL in volume and transferred onto a pad of Celite. After washing with pentane (12 mL), the residue was extracted into benzene. All volatiles were evaporated from the benzene solution in vacuo to afford **2b** as a red solid (46.3 mg, 42.5 μmol, 47%). Crystals suitable for X-ray diffraction were obtained by slow evaporation of saturated CH_2Cl_2 solutions of **2b** at –30°C, or by recrystallization from MeCN.

^{1}H NMR (500.1 MHz, CD_2Cl_2): δ = 0.85 [d, 12H, CH_3^{Dipp}, $^3J_{HH}$ = 6.44 Hz], 1.15 [d, 12H, CH_3^{Dipp}, $^3J_{HH}$ = 6.44 Hz], 1.36 [s, 12H, CH_3^{Caac}], 1.85 [s, 12H, CH_3^{Caac}], 1.98 [s, 4H, CH_2], 2.10 [s, 3H, $NCCH_3$], 2.88 [sept, 4H, $C^{Dipp}CH(CH_3)_2$, $^3J_{HH}$ = 6.44 Hz], 7.22 [virt d, 4H, m-$C^{Dipp}H$], 7.40 [virt t, 2H, o-$C^{Dipp}H$], 7.78 [4H, $B_2(CH)_4$].

^{13}C NMR (125.8 Hz, CD_2Cl_2): δ = 2.6 [$NCCH_3$], 25.4 [$C^{Carben}C(CH_3)_2$], 27.5 [($CH(CH_3)_2$)], 29.7 [$C^{Dipp}(CH(CH_3)_2)$], 32.5 [$NC(CH_3)_2$], 78.7 [$NC(CH_3)_2$], 126.3 [m-C^{Dipp}], 130.2 [p-C^{Dipp}], 136.0 [i-C^{Dipp}], 146.3 [o-C^{Dipp}], 151.2 [$B_2(CH)_4$].

^{11}B NMR (160.5 MHz, CD_2Cl_2): δ = 27.8.

IR (solid): 1371, 1387, 1423 (m, ν_{dbb} C=C), 1454 (s, ν_{cAAC} C-N), 2285 (w, ν_{MeCn} C-N), 2868, 2926, 2963 (m, ν_{dbb} C-H).

Elemental analysis calculated for $C_{46}H_{69}B_2Cl_4N_3Th$: C 52.14 H 6.56 N 3.97; Found: C 51.50 H 6.56 N 4.08.

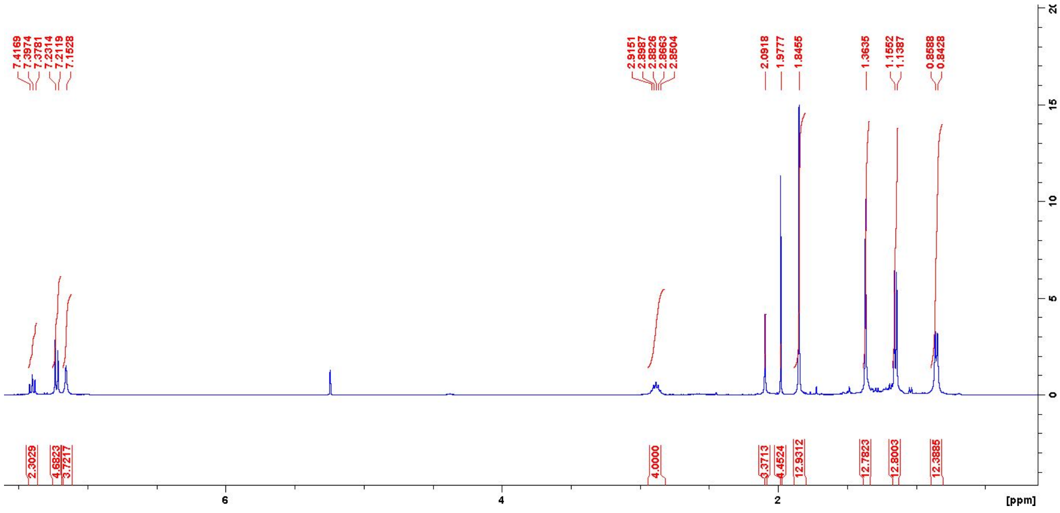

Figure S4. ^{1}H NMR spectrum of **2b** in CD_2Cl_2.

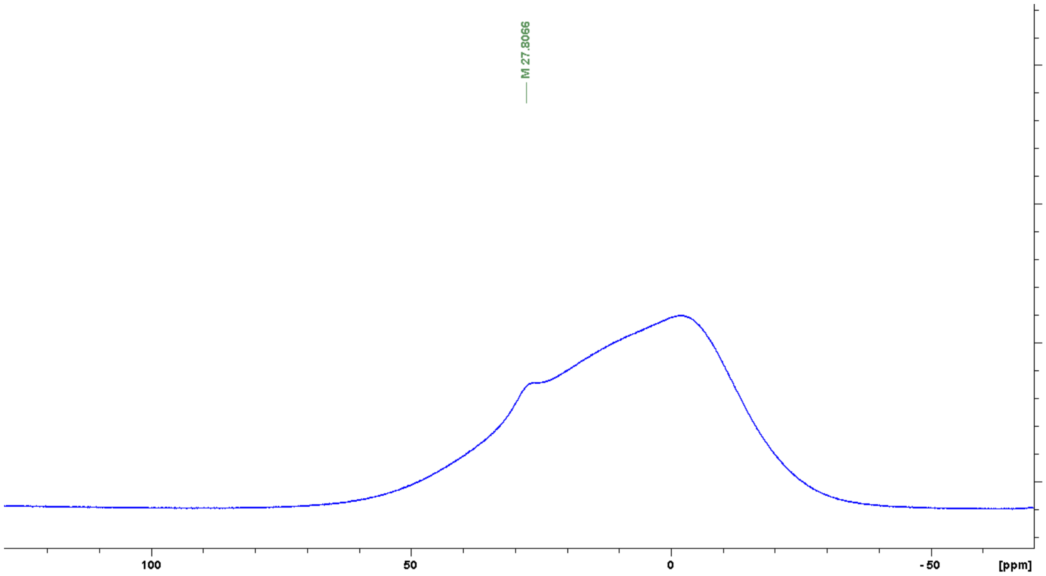

Figure S5. ^{11}B NMR spectrum of **2b** in CD_2Cl_2.

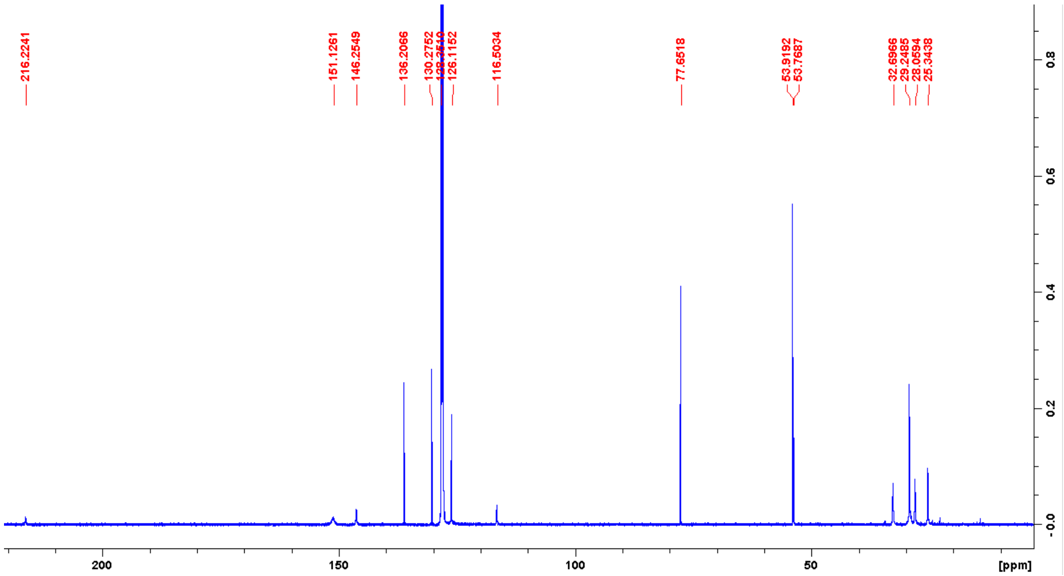

Figure S6. ^{13}C NMR spectrum of **2b** in CD_2Cl_2.

Synthesis and characterization of [(dbb)(PMe_3)$ThCl_4$] 2c

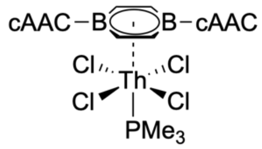

dbb (**1**) (55.4 mg, 86.0 μmol), and $ThCl_4(dme)_2$ (43.2 mg, 78.1 μmol) were suspended in benzene (0.5 mL), and treated with a solution of PMe_3 in hexanes (c = 320 μmol/L, 0.25 mL, 78.1 μmol). After heating at reflux over a period of 60 h, the heat source was turned off, and the red solution was slowly allowed to reach room temperature in the oil bath. During this process, small amounts of **2c** (9.4 mg, 8.6 μmol, 11%) formed as a red crystalline material, which was suitable for X-ray diffraction analysis. The crystals could be washed with aliphatic solvents, but **2c** is extremely sensitive to moisture and air, and readily decomposes under vacuum or in the presence of coordinating solvents.

^{1}H NMR (500.1 MHz, C_6D_6): δ = 1.02 [s, 12H, CH_3^{Caac}], 1.11 [d, 12H, CH_3^{Dipp}, $^3J_{\mathrm{HH}}$ = 6.75 Hz], 1.44 [d, 12H, CH_3^{Dipp}, $^3J_{\mathrm{HH}}$ = 6.16 Hz], 1.57 [s, 4H, CH_2], 1.86 [s, 12H, CH_3^{Caac}], 3.13 [s, 4H, $C^{Dipp}CH(CH_3)_2$], 7.07 [s, 2H, CH^{Dipp}], 7.09 [s, 3H, CH^{Dipp}], 7.14 [s, 2H, CH^{Dipp}], 7.69 [s, 4H, $B_2(CH)_4$].

^{11}B NMR (160.5 MHz, C_6D_6): δ = 27.7.

^{31}P NMR (202.4 MHz, C_6D_6): δ = –30.6.

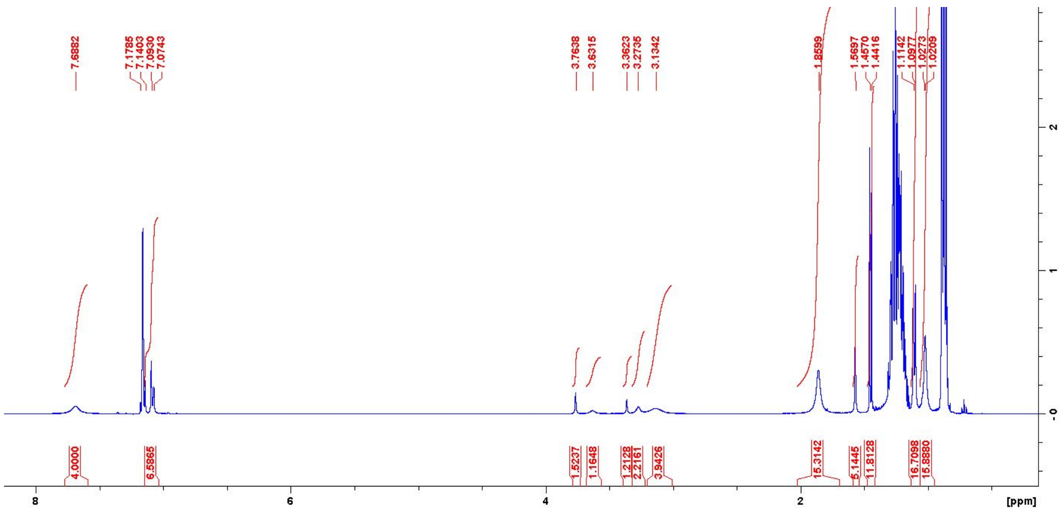

Figure S7. ^{1}H NMR spectrum of **2c** in C_6D_6.

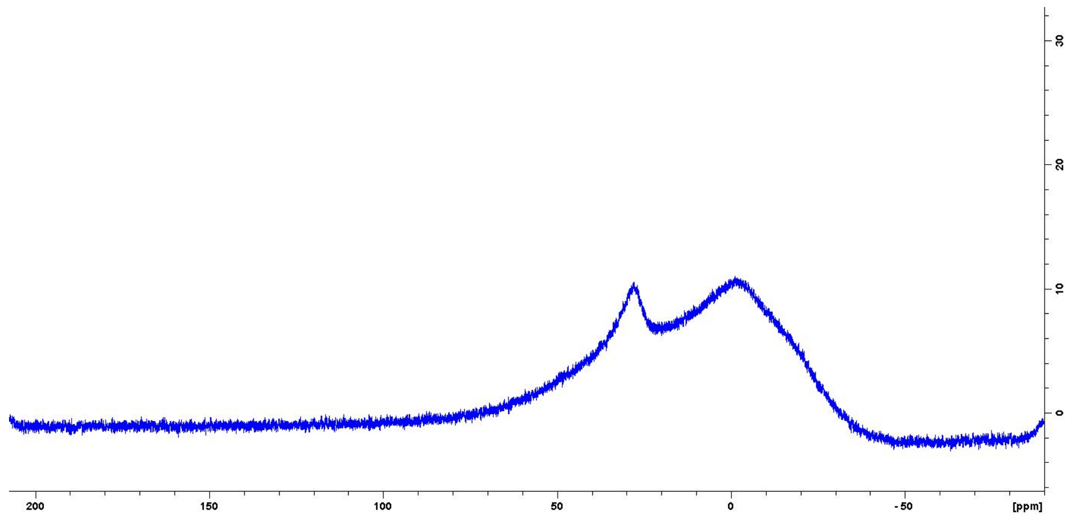

Figure S8. ^{11}B NMR spectrum of **2c** in C_6D_6.

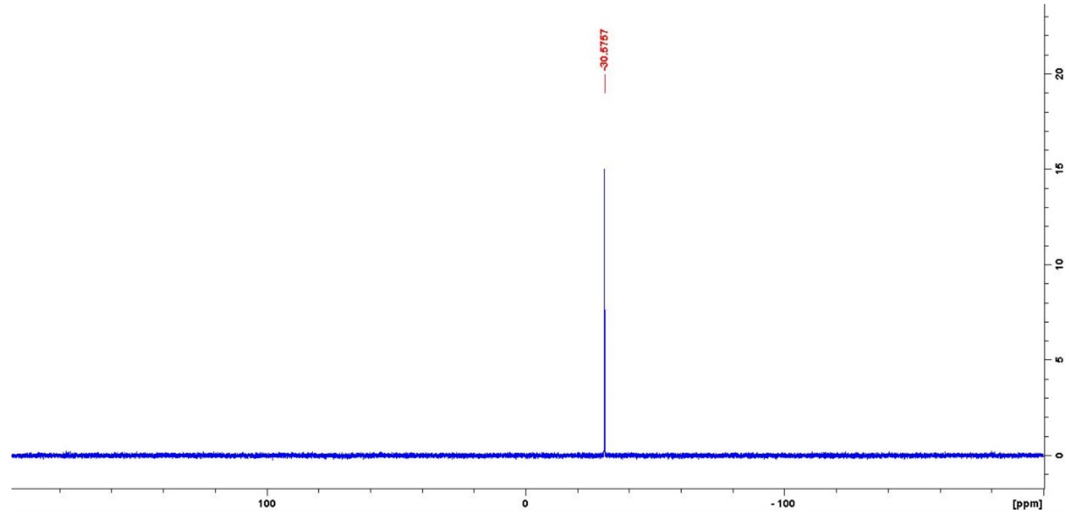

Figure S9. ^{31}P NMR spectrum of **2c** in C_6D_6.

Synthesis and characterization of [(dbb)(thf)UCl_4] 3a

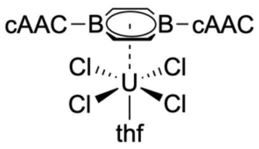

dbb (**1**) (373 mg, 580 μmol), and UCl_4 (200 mg, 527 μmol) were reacted in thf solution (10 mL) at reflux conditions over a period of 12 h, after which time the resulting deep red solution was allowed to slowly cool to room temperature. Thus, **3a** was isolated as deep red crystals, which were washed with pentane and dried in vacuo. Addition of pentane to the remaining thf solution afforded the precipitation of a second crop of **3a** (410 mg, 374 μmol, 71%). Crystals suitable for X-ray diffraction were obtained by slow evaporation of saturated CH_2Cl_2 solutions of **3a** containing few drops of benzene at −30°C.

^{11}B NMR (160.5 MHz, CD_2Cl_2): $\delta = -46.0$.

IR (solid): 1371, 1389 (m, ν_{dbb} C=C), 1456 (s, ν_{cAAC} C-N), 2869, 2930, 2966 (m, ν_{dbb} C-H).

Elemental analysis calculated for $C_{48}H_{74}B_2Cl_4ON_2U$: C 52.57 H 6.80 N 2.55; Found: C 51.97 H 6.74 N 2.52.

Evans NMR Method (CD_2Cl_2, rt): $\mu_{\text{eff}} = 2.61\ \mu_B$.

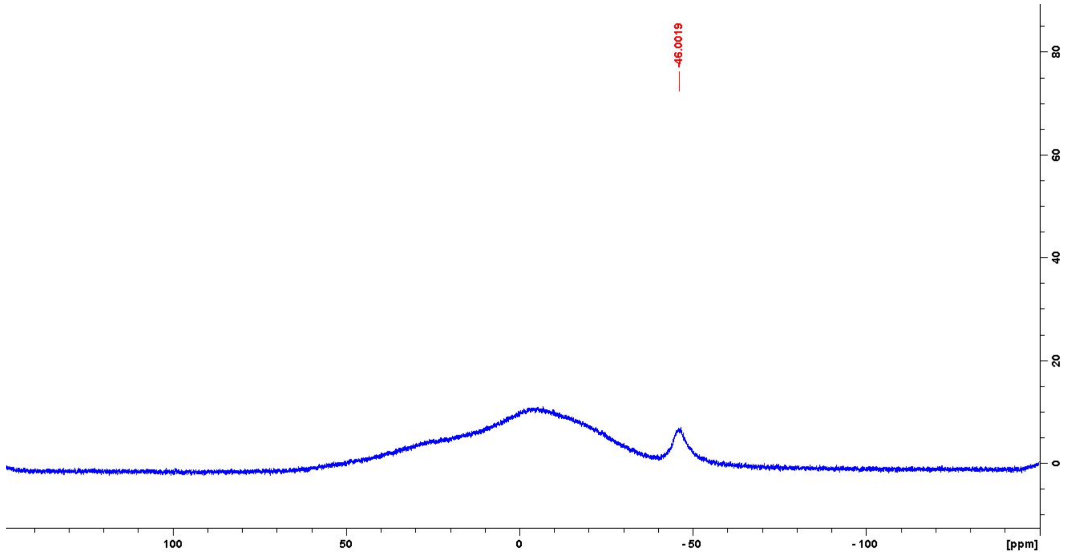

Figure S10. ^{11}B NMR spectrum of **3a** in CD_2Cl_2.

Synthesis and characterization of [(dbb)(MeCN)UCl_4] 3b

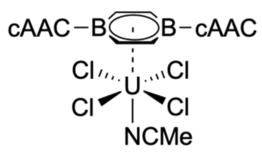

dbb (**1**) (187 mg, 290 μmol), and UCl_4 (100 mg, 263 μmol) were reacted in MeCN solution (6 mL) at reflux conditions over a period of 12 h, after which time the resulting deep red solution was allowed to slowly cool to room temperature. Thus, **3b** was isolated as deep red crystals, which were washed with pentane and dried in vacuo. Addition of pentane to the remaining MeCN solution afforded the precipitation of a second crop of **3b** (226 mg, 213 μmol, 81%). Crystals suitable for X-ray diffraction were obtained by slow evaporation of saturated CH_2Cl_2 solutions of **3b** at –30°C.

^{11}B NMR (160.5 MHz, CD_2Cl_2): δ = –70.0.

IR (solid): 1372, 1388 (m, ν_{dbb} C=C), 1457 (s, ν_{cAAC} C-N), 2869, 2931, 2967 (m, ν_{dbb} C-H).

Elemental analysis calculated for $C_{48}H_{72}B_2Cl_4N_4U$: C 52.09 H 6.56 N 5.06; Found: C 52.27 H 6.76 N 4.68.

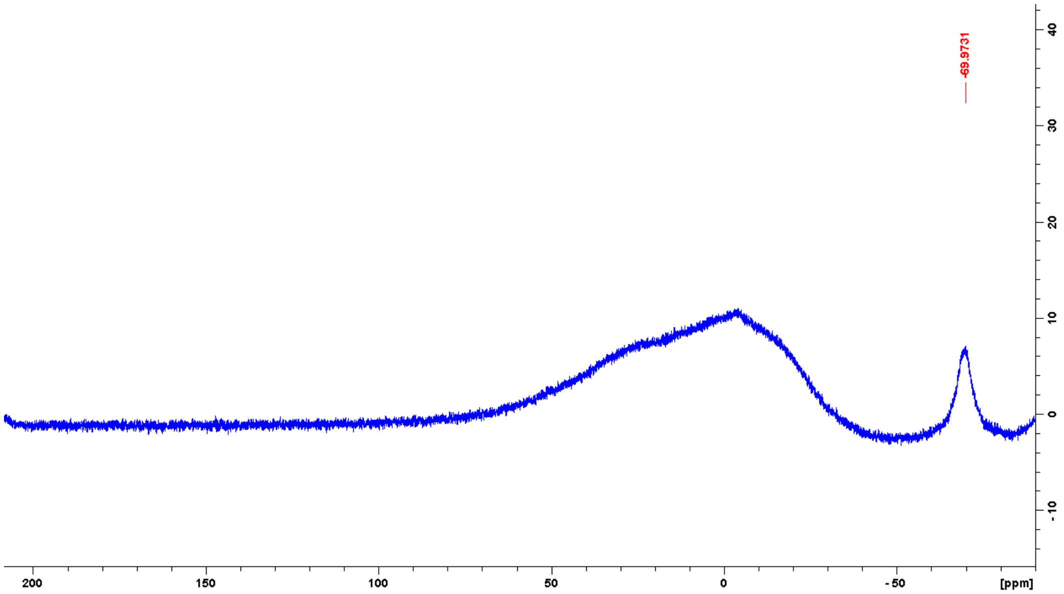

Figure S11. ^{11}B NMR spectrum of **3b** in CD_2Cl_2.

Synthesis and characterization of [{(dbb)$ThCl_4$}$_2$-*κ*-dme] 4

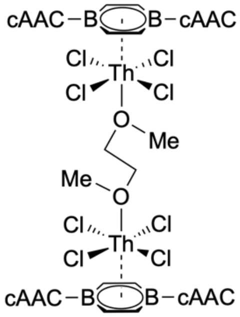

dbb (**1**) (64.0 mg, 99.3 *μ*mol), and $ThCl_4(dme)_2$ (50.0 mg, 90.0 *μ*mol) were suspended in fluorobenzene (5 mL) at reflux conditions over a period of 16 h. The resulting purple reaction mixture was filtered over Celite, and concentrated in vacuo to about 1 mL. Addition of hexane (5 mL) caused the precipitation of a red solid, which was collected and dried in vacuo to afford **4** (20.0 mg, 9.5 *μ*mol, 10%). Crystals suitable for X-ray diffraction were obtained by slow evaporation of saturated CH_2Cl_2 solutions of **4**.

^{11}B NMR (160.5 MHz, CD_2Cl_2): $\delta = -27.8$.

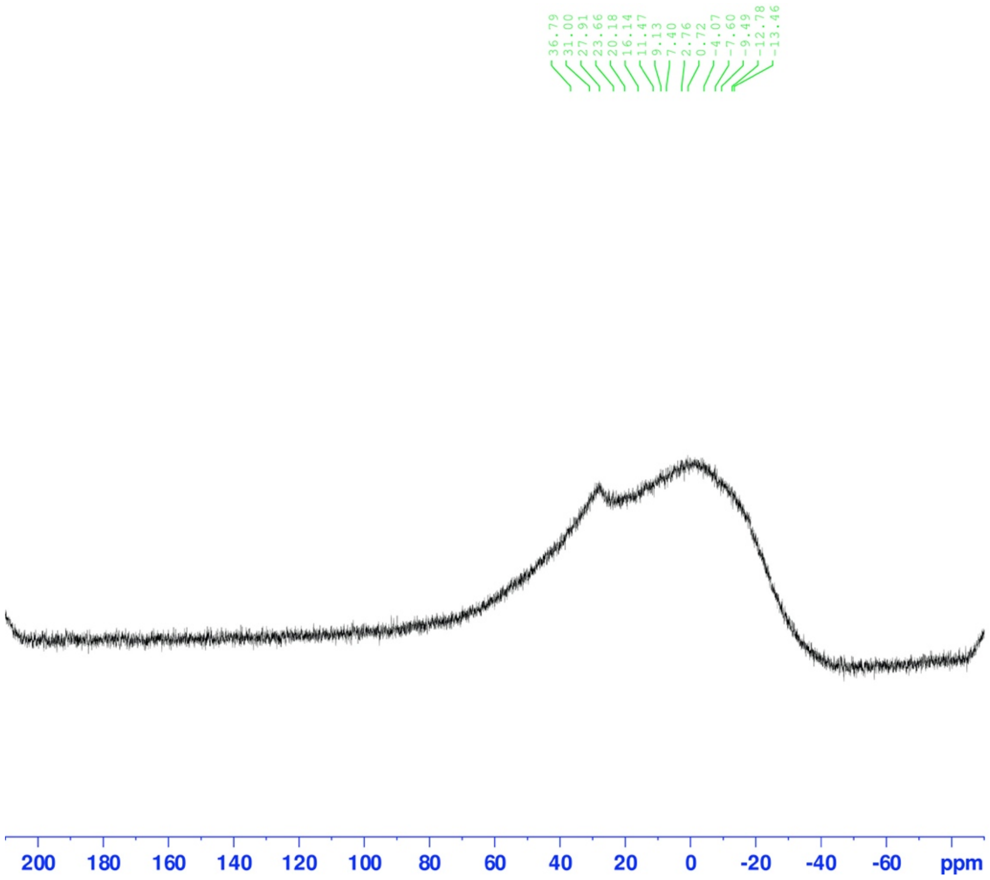

Figure S12. ^{11}B NMR spectrum of **3b** in CD_2Cl_2.

S2 IR spectroscopy

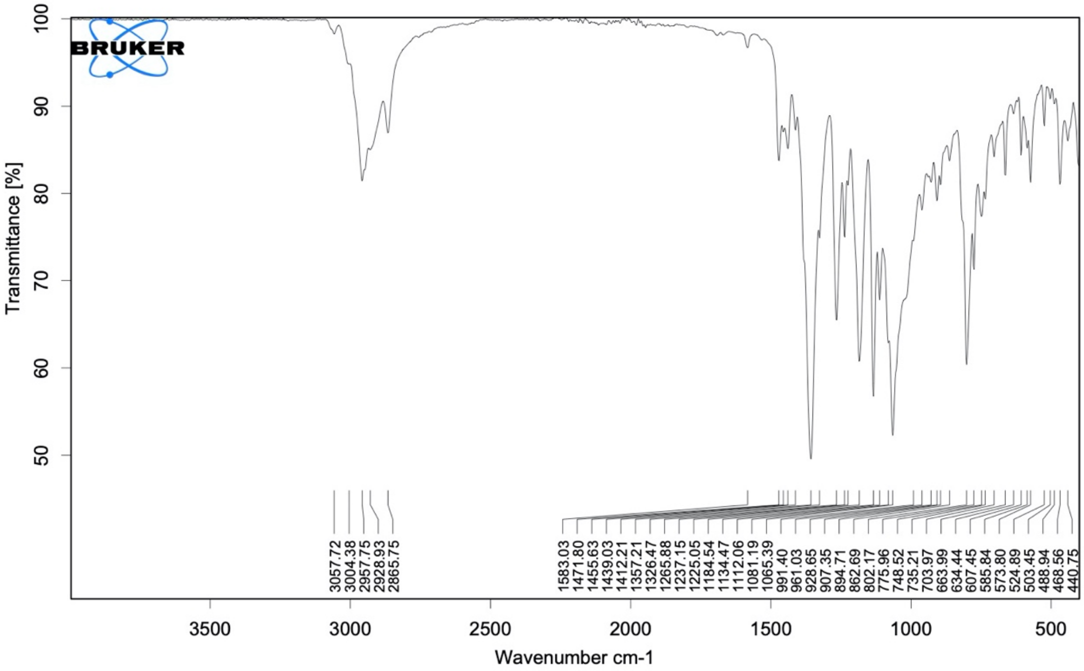

Figure S13. IR spectrum of **1**.

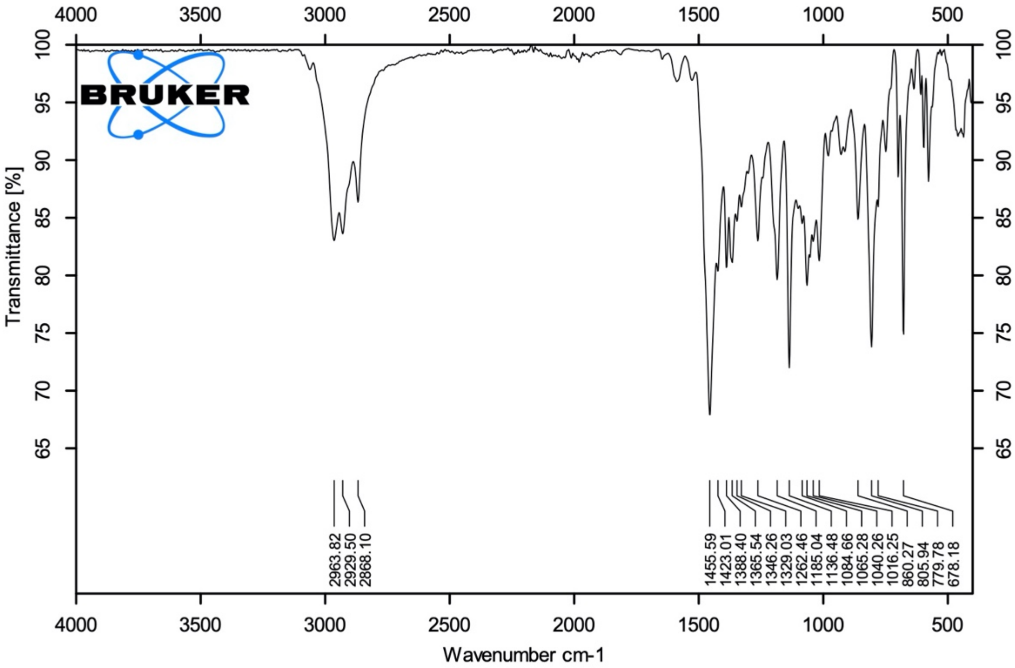

Figure S14. IR spectrum of **2a**.

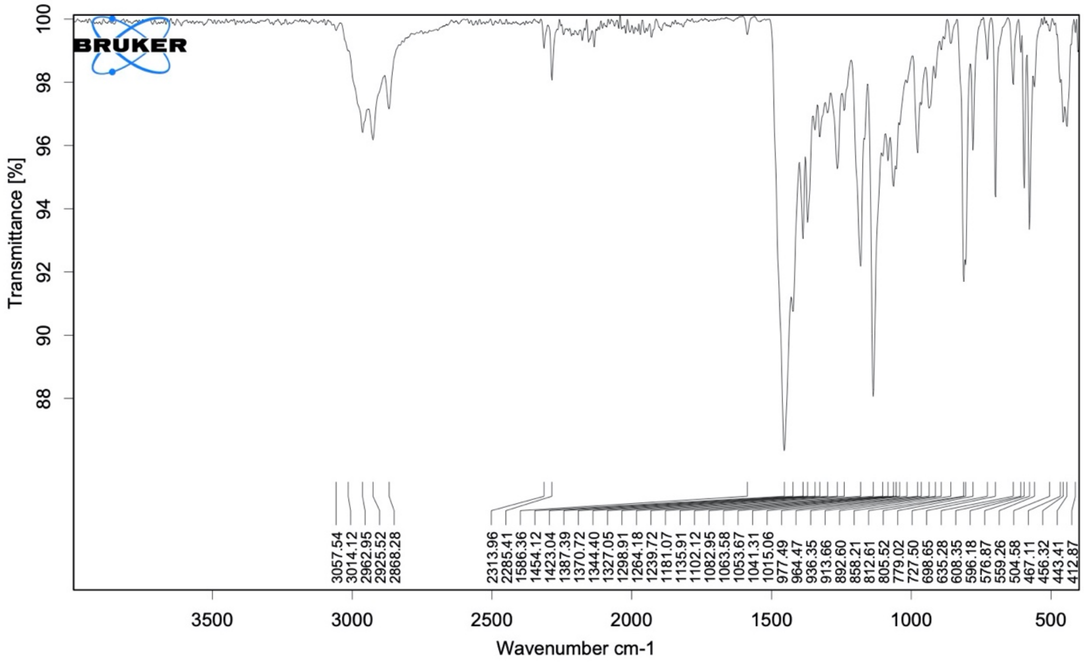

Figure S15. IR spectrum of **2b**.

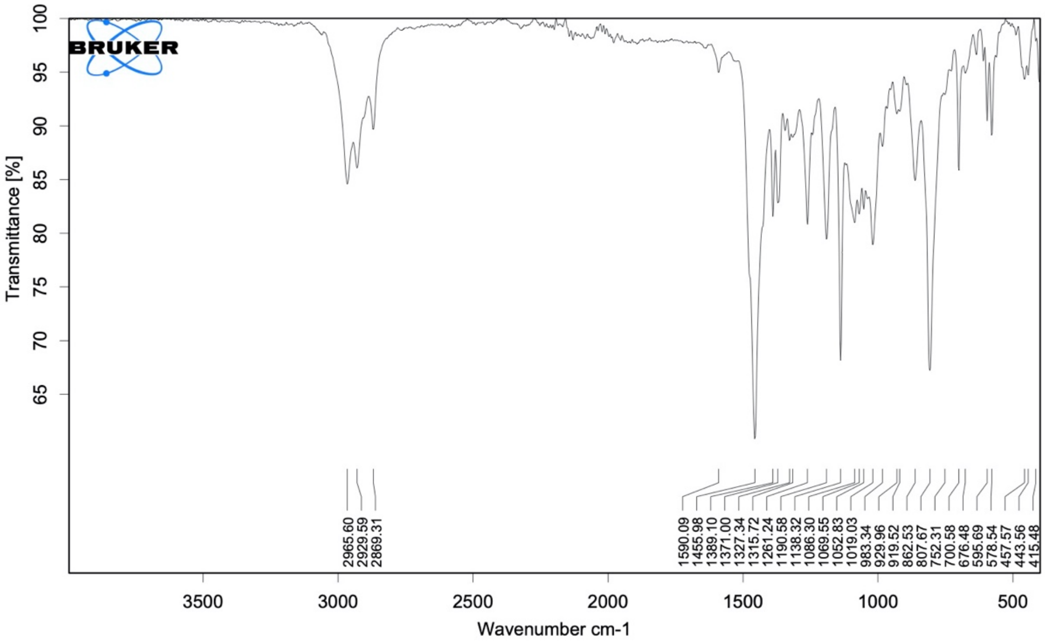

Figure S16. IR spectrum of **3a**.

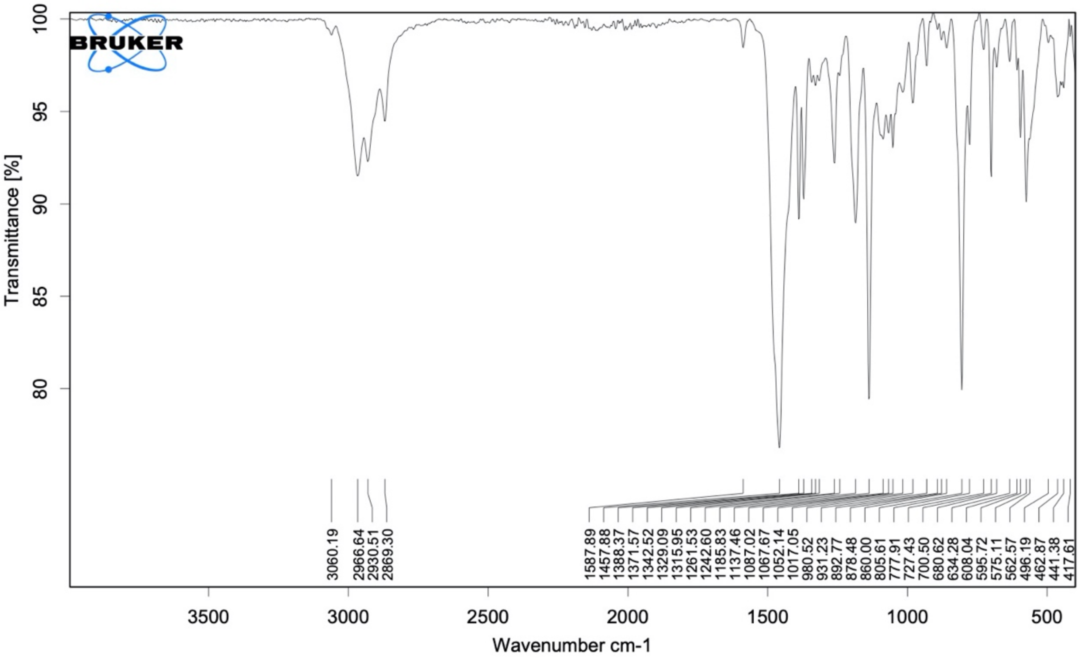

Figure S17. IR spectrum of **3b**.

S3 X-ray diffraction data

General remarks: The crystal data of **2a**, **2b** and **2c** were collected on a BRUKER SMART-APEX diffractometer with a CCD area detector and graphite monochromated $Mo_{K\alpha}$ radiation. The crystal data of **3a**, **3b** and **4** were collected on a BRUKER X8-APEX II diffractometer with a CCD area detector and multi-layer mirror monochromated $Mo_{K\alpha}$ radiation. The structures were solved using intrinsic phasing method (SHELXT),[4] refined with the SHELXL program,[5] and expanded using Fourier techniques. All non-hydrogen atoms were refined anisotropically. Hydrogen atoms were included in structure factors calculations. All hydrogen atoms were assigned to idealized geometric positions.

Crystal data for **2a**: $C_{48}H_{74}B_2Cl_4ON_2Th$ $M_r = 1246.32$, red plate, $0.13\times0.05\times0.01$ mm^3, Monoclinic space group $P2_1/c$, $a = 25.0547(11)$ Å, $b = 10.6023(5)$ Å, $c = 23.0031(11)$ Å, $\beta = 107.421(2)°$, $V = 5830.2(5)$ Å^3, $Z = 4$, $\rho_{calcd} = 1.420$ g·cm^{-3}, $\mu = 2.781$ mm^{-1}, $F(000) = 2542$, $T = 296(2)$ K, $R_1 = 0.0559$, $wR^2 = 0.0837$, 11470 independent reflections [$2\theta \leq 52.042°$] and 710 parameters. Due to severe disorder, the atomic displacement parameters of atoms C5-C12 (2nd cAAC), C13-C23 (Dipp-substituent of 2nd cAAC), and C45-C48 (thf) were restrained using SIMU, RIGU, and DELU keywords in ShelXL input ('rigid bond' restraint for the bonds in the connectivity list. Standard values of 0.004 for both parameters s1 and s2 were used). The asymmetric unit contains two molecules of co-crystallized benzene.

Crystal data for **2b**: $C_{50}H_{75}B_2Cl_4N_5Th$, $M_r = 1141.61$, red plate, $0.43\times0.10\times0.029$ mm^3, Triclinic space group $P\bar{1}$, $a = 10.3321(9)$ Å, $b = 15.8242(14)$ Å, $c = 18.2791(16)$ Å, $\alpha = 67.153(3)°$, $\beta = 78.842(3)°$, $\gamma = 80.653(3)°$, $V = 2689.4(4)$ Å^3, $Z = 2$, $\rho_{calcd} = 1.410$ g·cm^{-3}, $\mu = 3.008$ mm^{-1}, $F(000) = 1156$, $T = 100(2)$ K, $R_1 = 0.0268$, $wR^2 = 0.0558$, 10597 independent reflections [$2\theta \leq 52.044°$] and 578 parameters. The asymmetric unit contains two molecules of co-crystallized acetonitrile.

Crystal data for **2c**: $C_{56}H_{84}B_2Cl_4N_2PTh$, $M_r = 1211.68$, red plate, $0.19\times0.07\times0.02$ mm^3, Monoclinic space group $P2_1/c$, $a = 23.4398(9)$ Å, $b = 11.6969(5)$ Å, $c = 23.6865(10)$ Å, $\beta = 116.3240(10)°$, $V = 5820.8(4)$ Å^3, $Z = 4$, $\rho_{calcd} = 1.383$ g·cm^{-3}, $\mu = 2.809$ mm^{-1}, $F(000) = 2468$, $T = 100(2)$ K, $R_1 = 0.0604$, $wR^2 = 0.0726$, 11468 independent reflections [$2\theta \leq 52.044°$] and 584 parameters. The asymmetric unit contains two molecules of co-crystallized benzene. The displacement parameters of atoms C60-C66 (co-crystallized benzene molecule) were restrained to the same value with similarity restraint (SIMU).

Crystal data for **3a**: $C_{60}H_{86}B_2Cl_4N_2OU$, $M_r = 1252.75$, red plate, $0.13\times0.05\times0.01$ mm^3, Monoclinic space group $P2_1/c$, $a = 25.0547(11)$ Å, $b = 10.6023(5)$ Å, $c = 23.0031(11)$ Å, $\beta = 107.421(2)°$, $V = 5830.2(5)$ Å^3, $Z = 4$, $\rho_{calcd} = 1.427$ g·cm^{-3}, $\mu = 3.008$ mm^{-1}, $F(000) = 2552$, $T = 100(2)$ K, $R_1 = 0.0569$, $wR^2 = 0.0885$, 11477 independent reflections [$2\theta \leq 52.042°$] and 707 parameters. The displacement parameters of atoms C5-C12 (2nd cAAC), C13-C23 (Dipp-substituent of 2nd cAAC), and C45-C48 (thf) were restrained to the same value with similarity restraint (SIMU) due to disorder. The asymmetric unit contains two molecules of co-crystallized benzene.

Crystal data for **3b**: $C_{46}H_{69}B_2Cl_4N_3U$, M_r = 1065.49, red plate, 0.095×0.056×0.01 mm^3, Triclinic space group $P\bar{1}$, a = 10.3481(17) Å, b = 15.835(3) Å, c = 18.246(3) Å, α = 66.374(6)°, β = 78.138(6)°, γ = 79.066(6)°, V = 2661.5(8) Å^3, Z = 2, ρ_{calcd} = 1.330 g·cm^{-3}, μ = 3.281 mm^{-1}, F(000) = 1072, T = 296(2) K, R_1 = 0.0234, wR^2 = 0.0467, 10608 independent reflections [2θ≤52.308°] and 525 parameters. The unit cell contains two molecules of acetonitrile, which have been treated as a diffuse contribution to the overall scattering without specific atom positions by SQUEEZE/PLATON.[6]

Crystal data for **4**: $C_{92}H_{150}B_4Cl_8N_4O_2Th_2$, M_r = 2135.17, red plate, Monoclinic space group $P2_1/c$, a = 15.7048(12) Å, b = 11.5529(8) Å, c = 30.981(2) Å, β = 93.050(3)°, V = 5613.1(7) Å^3, Z = 4, F(000) = 2388, T = 100(2) K, R_1 = 0.0422, wR^2 = 0.0973, 11037 independent reflections [2θ≤52.044°] and 660 parameters. The quality of this data set was too poor for any discussion of bond parameters.

Crystallographic data have been deposited for **2a**, **2b**, **2c**, **3a**, and **3b** with the Cambridge Crystallographic Data Center as supplementary publication no. CCDC1940751-1940755. These data can be obtained free of charge from The Cambridge Crystallographic Data Centre *via* www.ccdc.cam.ac.uk/data_request/cif.

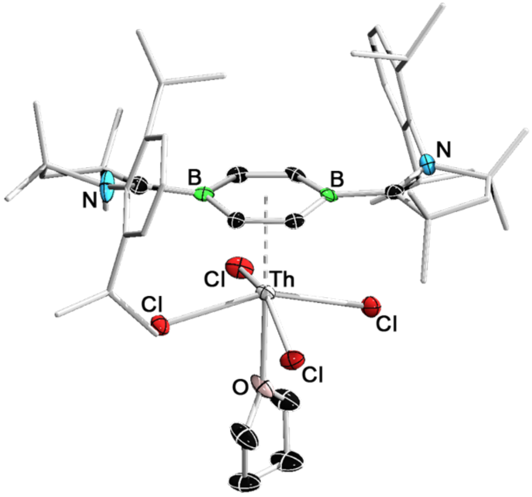

Figure S18. Molecular structure of **2a** in the solid state. Thermal displacement parameters are displayed at the 50%-probability level. Hydrogen atoms, most ellipsoids of the cAAC-substituents, and co-crystallized solvent molecules are omitted for clarity. Selected bond lengths (Å): Th-Cl 2.695 (av.), Th-O 2.524(3), Th-C_{dbb} 2.929(4)-2.948(4), Th-dbb_{cent} 2.586, Th-B 3.043(5), 3.055(6), B-C_{cAAC} 1.584(6), 1.591(6).

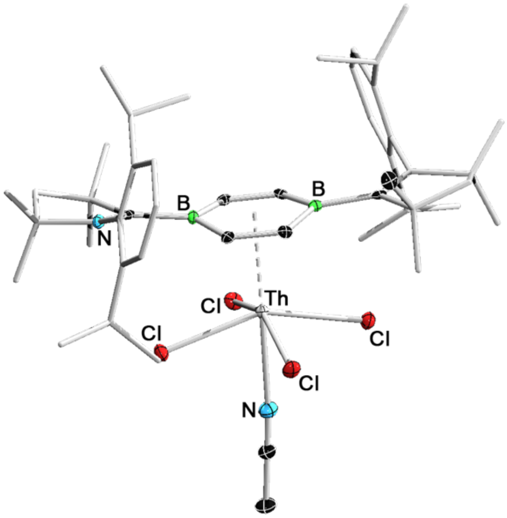

Figure S19. Molecular structure of **2b** in the solid state. Thermal displacement parameters are displayed at the 50%-probability level. Hydrogen atoms, most ellipsoids of the cAAC-substituents, and co-crystallized solvent molecules are omitted for clarity. Selected bond lengths (Å): Th-Cl 2.690 (av.), Th-N 2.596(3), Th-C_{dbb} 2.880(2)-2.945(3), Th-dbb_{cent} 2.556, Th-B 3.018(3), 3.054(3), B-C_{cAAC} 1.591(4), 1.597(4).

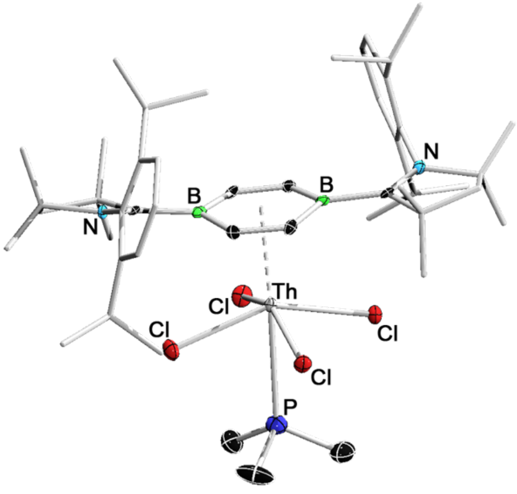

Figure S20. Molecular structure of **2c** in the solid state. Thermal displacement parameters are displayed at the 50%-probability level. Hydrogen atoms, most ellipsoids of the cAAC-substituents, and co-crystallized solvent molecules are omitted for clarity. Selected bond lengths (Å): Th-Cl 2.690 (av.), Th-P 3.053(2), Th-C_{dbb} 2.890(4)-2.953(4), Th-dbb_{cent} 2.557, Th-B 3.019(4), 3.020(4), B-C_{cAAC} 1.587(6), 1.591(6).

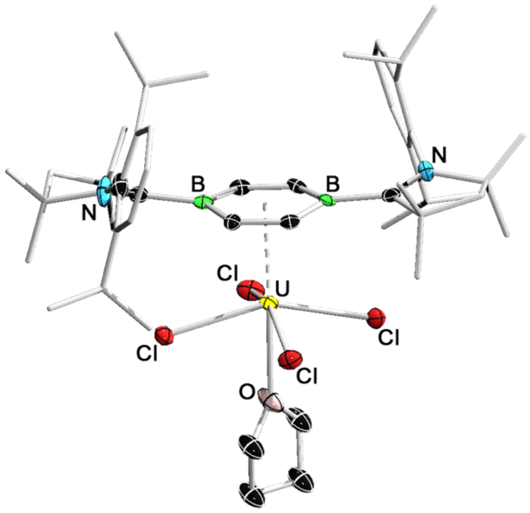

Figure S21. Molecular structure of **3a** in the solid state. Thermal displacement parameters are displayed at the 50%-probability level. Hydrogen atoms, most ellipsoids of the cAAC-substituents, and co-crystallized solvent molecules are omitted for clarity. Selected bond lengths (Å): U-Cl 2.694 (av.), U-O 2.523(3), U-C_{dbb} 2.930(4)-2.947(4), U-dbb_{cent} 2.585, U-B 3.041(5), 3.055(5), B-C_{cAAC} 1.586(7), 1.591(7).

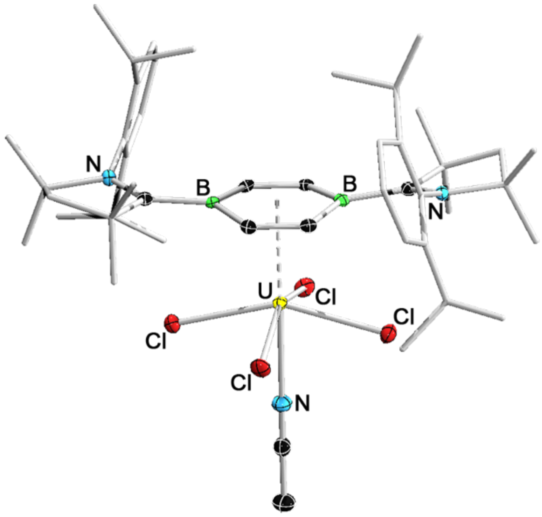

Figure S22. Molecular structure of **3b** in the solid state. Thermal displacement parameters are displayed at the 50%-probability level. Hydrogen atoms, and most ellipsoids of the cAAC-substituents are omitted for clarity. Selected bond lengths (Å): U-Cl 2.633 (av.), U-N 2.539(2), U-C_{dbb} 2.831(2)-2.877(2), U-dbb_{cent} 2.490, U-B 2.962(2), 2.997(2), B-C_{cAAC} 1.589(3), 1.590(3).

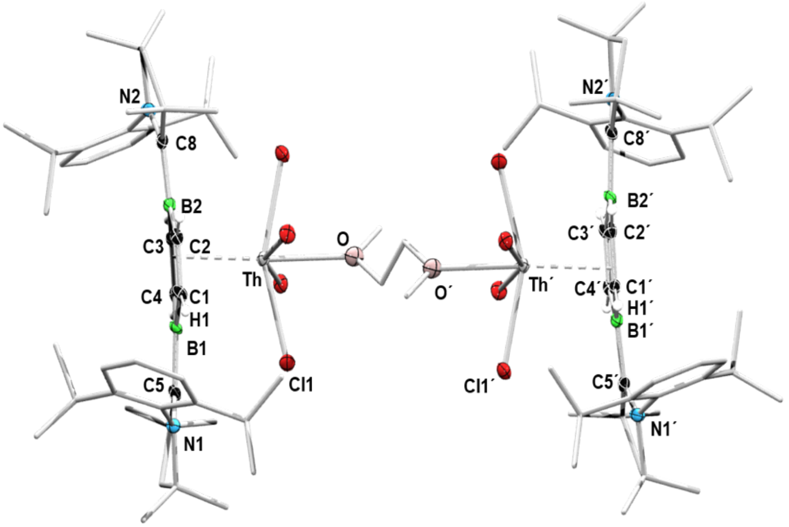

Figure S23. Molecular structure of **4** in the solid state. Thermal displacement parameters are displayed at the 50%-probability level. Hydrogen atoms, and most ellipsoids of the cAAC-substituents are omitted for clarity. The quality of the crystallographic data set was too poor to allow for a discussion of bonding parameters.

S4 SQUID magnetization data

General remarks: Magnetic susceptibility measurements were completed with a MPMS-XL7 Quantum Design SQUID magnetometer. Direct current (dc) susceptibility data measurements were performed at temperatures ranging from 1.8 K to 300 K, and applied fields up to 7 T. 20.0 mg (**3a**, **3b**), and 21.0 mg (UCl_4) of crushed polycrystalline samples wrapped in a polyethylene membrane were used. Magnetization *vs.* field measurements were performed at 100 K in order to check for the presence of ferromagnetic impurities, which were found to be absent. Magnetic data was corrected for diamagnetic contributions using Pascal's constants.

Discussion: SQUID magnetization data of **3a/b** in the solid state are consistent with $5f^2d^0$ ($^3H_4$) electron configurations (Figures 2, S24 and S15). Accordingly, μ_{eff} gradually decreases from 2.685 μ_B (**3a**) and 2.840 μ_B (**3b**) at 300 K, followed by a rapid decrease below 50 K to values of 0.371 μ_B (**3a**) and 0.488 μ_B (**3b**) at 1.8 K, resulting in a curvature reminiscent for U(IV) complexes. Such behavior is indicative of a singlet ground state with temperature-independent paramagnetism at very low temperatures.[7] This correlates with the low temperature isothermal magnetization curves, which display small temperature-independent magnetic moments (< 2.0 μ_B). Plots of χT *vs.* T show a clear and precipitous decrease in the susceptibility below 100 K, nearing 0.0 $cm^3\ K\ mol^{-1}$, clearly indicating the singlet ground state (Figure S24). Notably, the high temperature magnetic moments of **3a/b** are smaller than that obtained for UCl_4 (3.25 μ_B at 300 K) under the same conditions. Strong ligand fields/strongly donating ligands may quench the orbital contribution to the magnetic moment due to covalency effects in 5*f* ions, reducing the observed magnetic moment.[8] This leads to the characteristically low magnetization values for **3a/b** when compared to the statistical average of all U(IV) complexes (2.77 μ_B, $\sigma = 0.39$), as well as UCl_4.[9]

Table S1. Experimentally determined values of the effective magnetic moment (μ_{eff}) and the magnetic susceptibility (χT) of the U(IV) compounds **3a**, **3b**, and UCl_4.

	**3a**	**3b**	UCl_4	T [K]
μ_{eff} [μ_B]	2.685	2.840	3.252	300
	0.371	0.488	0.527	1.8
χT [$cm^3\ K\ mol^{-1}$]	0.901	1.00	1.322	300
	0.017	0.029	0.035	1.8

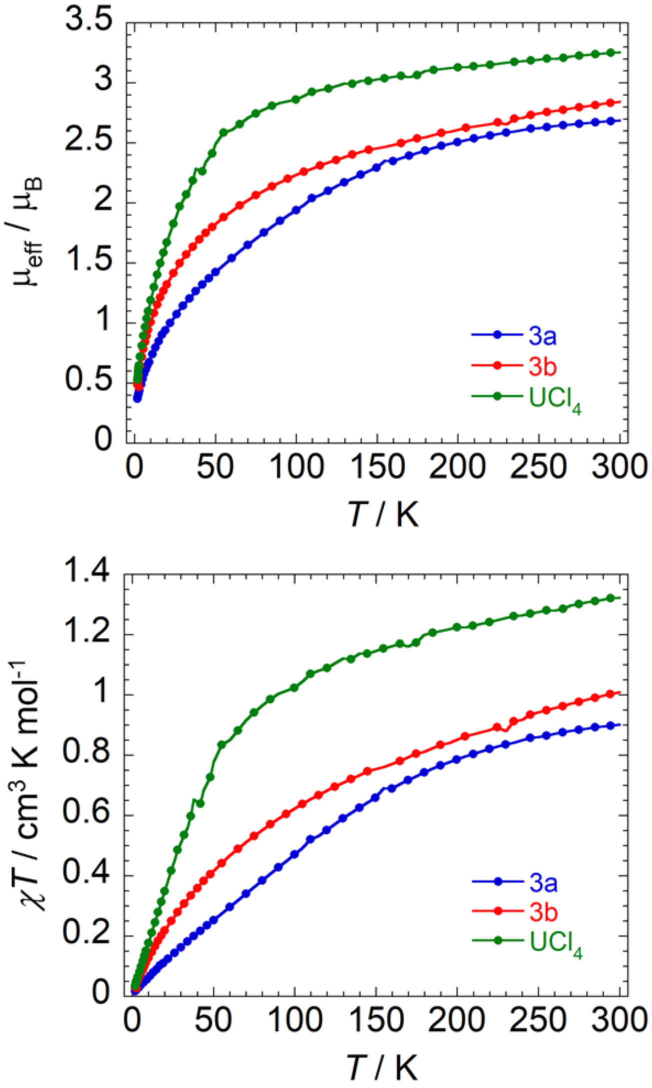

Figure S24. Temperature-dependent SQUID magnetization data (at H = 1000 Oe) of U(IV) complexes **3a**, **3b** and UCl_4, as a function of the effective magnetic moment (μ_{eff}; *top*) and the magnetic susceptibility (χT; *bottom*) *vs*. the temperature (T).

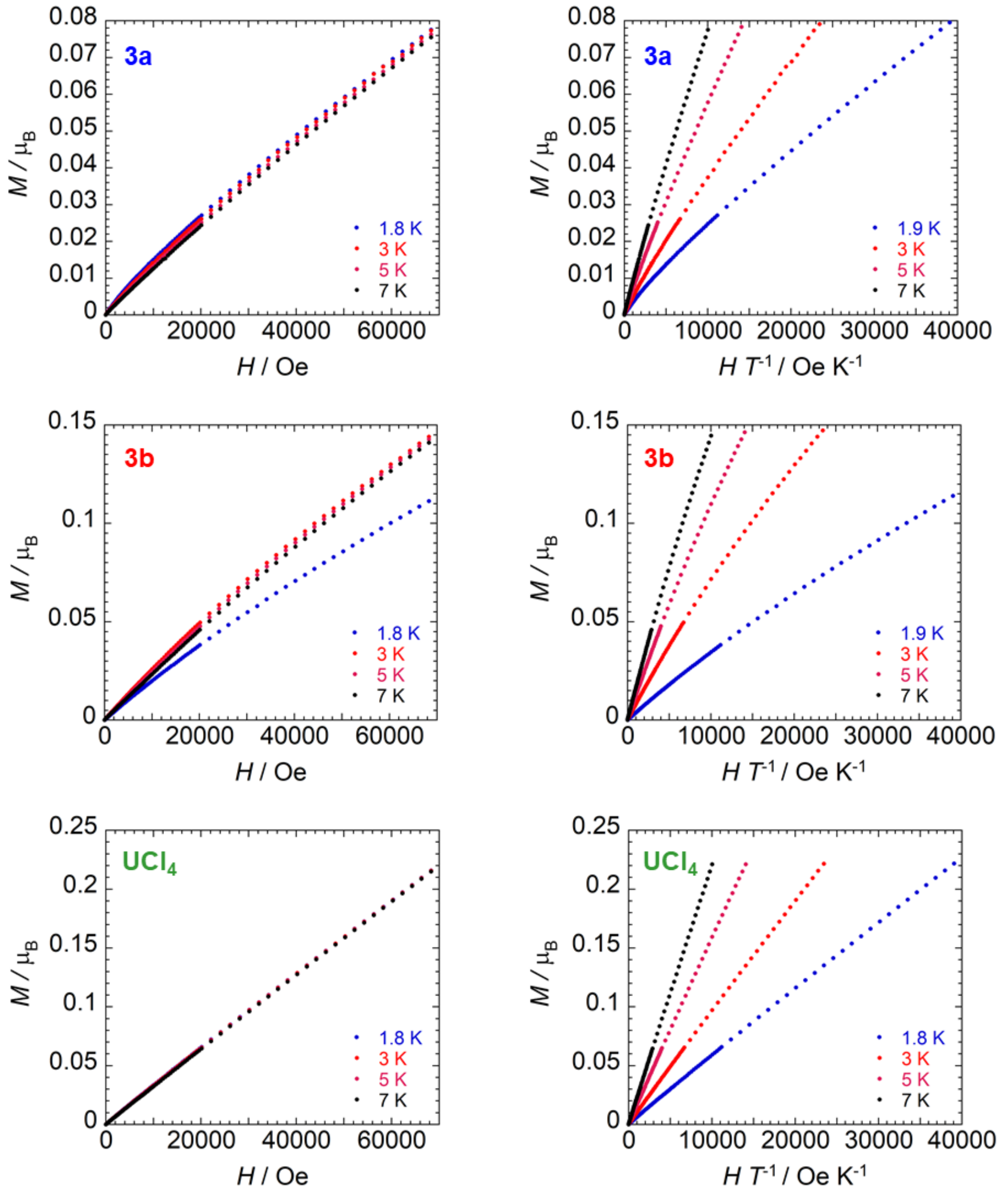

Figure S25. Solid-state field dependence (H = 0-70 kOe) of the magnetization (*left*) and reduced magnetization (*right*) for **3a** (*top*), **3b** (*middle*), and UCl_4 (*bottom*) at the indicated temperatures (T).

S5 Computational details

General remarks: All structures were fully optimized at the PBE0 level of theory,[10] including an atom-pairwise correction for dispersion forces *via* Grimme's D3 model[11] with Becke-Johnson (BJ)[12] damping in the Turbomole program.[13] Quasirelativistic energy-consistent small-core pseudopotentials (effective-core potentials, ECP)[14] with corresponding (14s13p10d8f1g)/[10s9p5d4f1g] Gaussian-type orbital (GTO) valence basis sets were used for actinides, whereas ligand atoms were treated with an all-electron def2-TZVP basis set.[15] The PBE0-D3(BJ)/def2-TZVP/ECP optimized structures were compared to available X-ray data (Table S2) and subsequently used for electronic structure analysis and NMR shift calculations.

Relativistic all-electron DFT calculations of the nuclear shieldings were performed using the Amsterdam Density Functional (ADF) program suite,[16] employing the PBE0 exchange-correlation functional[10] in conjunction with Slater-type orbital basis sets of triple-ζ doubly polarized (TZ2P) quality and an integration accuracy of 5.0. The calculations used gauge-including atomic orbitals (GIAOs). The ZORA calculations of NMR chemical shifts[17] were done with the previously neglected terms from the exchange–correlation (XC) response kernel,[18] which were shown to be important for systems with large SO shift contributions, such as actinide complexes.[19] The computed ^{1}H, ^{13}C and ^{11}B nuclear shieldings were converted to chemical shifts (δ, in ppm) relative to the shieldings of TMS and $BF_3 \cdot OEt_2$ (δ = 0.0 ppm) as references, respectively. In the case of ^{11}B, we considered B_2H_6 as a secondary standard, with δ_B = 16.6 ppm *vs.* $BF_3 \cdot OEt_2$.[20] Both reference compounds were computed at the same level as used for the actinide complexes (2c-ZORA/PBE0/TZ2P).

Natural population analyses (NPA)[21] and Bader's QTAIM (quantum theory of atoms-in-molecules) analyses[22] of the Kohn-Sham (KS) wave functions, generated in Gaussian 09[23] at the same level as used in the structure optimization (PBE0-D3(BJ)/def2-TZVP/ECP) and stored as .wfx files, were performed using the Multiwfn program.[24] The latter code was also employed for calculation of oxidation states in actinide complexes using a localized orbital bonding analysis (LOBA).[25] In the QTAIM analyses, we focused particularly on the delocalization indices (DI) as a measure of the bond covalency. The DI integrates the electron density in the bonding region between two atoms in question and is closely related to the covalent bond order, reduced by bond polarity (*i.e.*, DI = 1.0 for a "pure" covalent single bond, but DI = 0.0 for a "pure" ionic bond).

The strength of the actinide···arene interactions was evaluated using a quantitative energy decomposition analysis (EDA)[26] of the total bonding energy into electrostatic interactions, Pauli-repulsive orbital interactions, and attractive orbital (covalent) interactions, as implemented in the ADF code. For technical reasons, open-shell U(III) and U(IV) fragments were treated first in a spin restricted single-point calculation, followed by an unrestricted single point SCF at the same structure with the FragOccupations input keyword allowing to specify a proper occupation of alpha and beta orbitals and accompanied by EDA analysis. This provided a correction energy term, which was substracted from the orbital and total bonding energies computed for open-shell arene complexes in the unrestricted mode, again employing the restricted fragments along with the FragOccupations option for both arene and radical U(III)/U(IV) fragments.[27]

In addition, the Kohn-Sham wave functions were analyzed by means of the electron localization function (ELF)[28], using the DGrid program[29] with grids using ten points per Bohr. The results of ELF analyses were visualized using the ParaView program.[30]

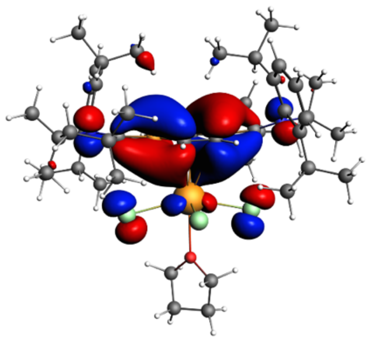

HOMO
4% Th(d)
2 x 18% B(p)
4 x 6% C_{dbb}(p)
2 x 3% C_{CAAC}(p)
2 x 4% N_{CAAC}(p)
2 x 4% Cl(p)

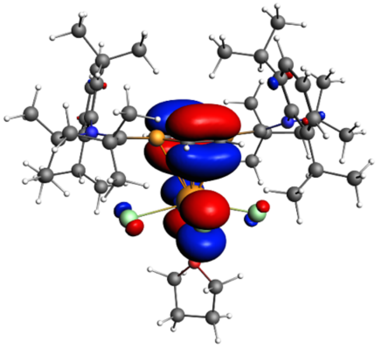

HOMO-1
3% Th(f)
4 x 14% C_{dbb}(p)
2 x 16% Cl(p)
2 x 2% Cl(p)

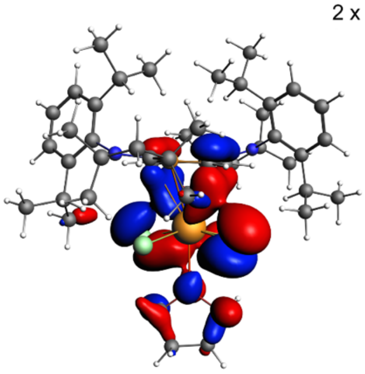

HOMO-16
11% Th(d)
4 x 4% C_{dbb}(p)
2 x 19% Cl(p)
2 x 5% Cl(p)
5% O(p)

Figure S26. Frontier molecular orbital representations of **2a** relevant to the actinide-arene bonding (isosurface plots ± 0.03 a.u.).

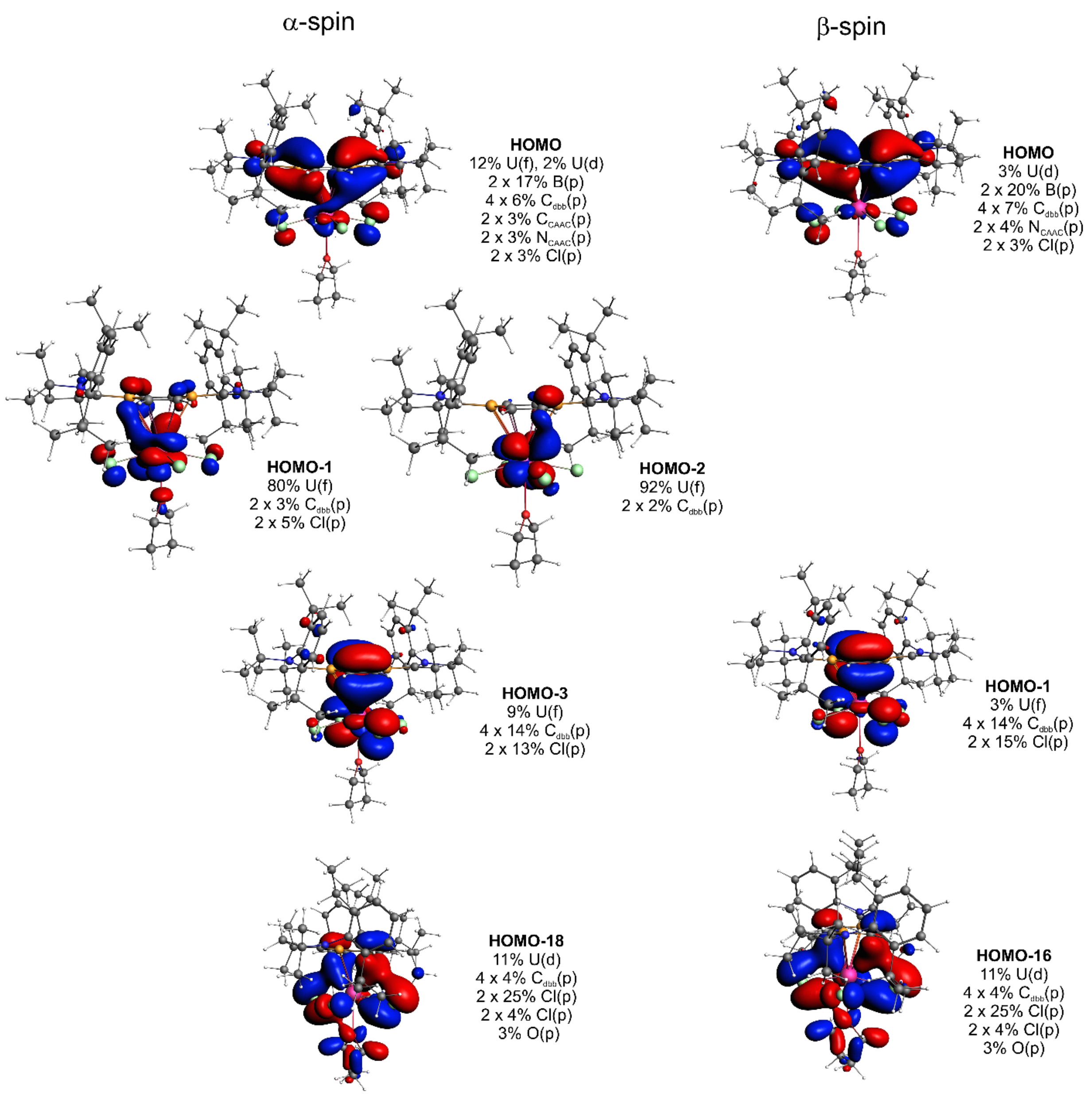

Figure S27. Frontier molecular orbital representations of **3a** relevant to the actinide-arene bonding (isosurface plots ± 0.03 a.u.).

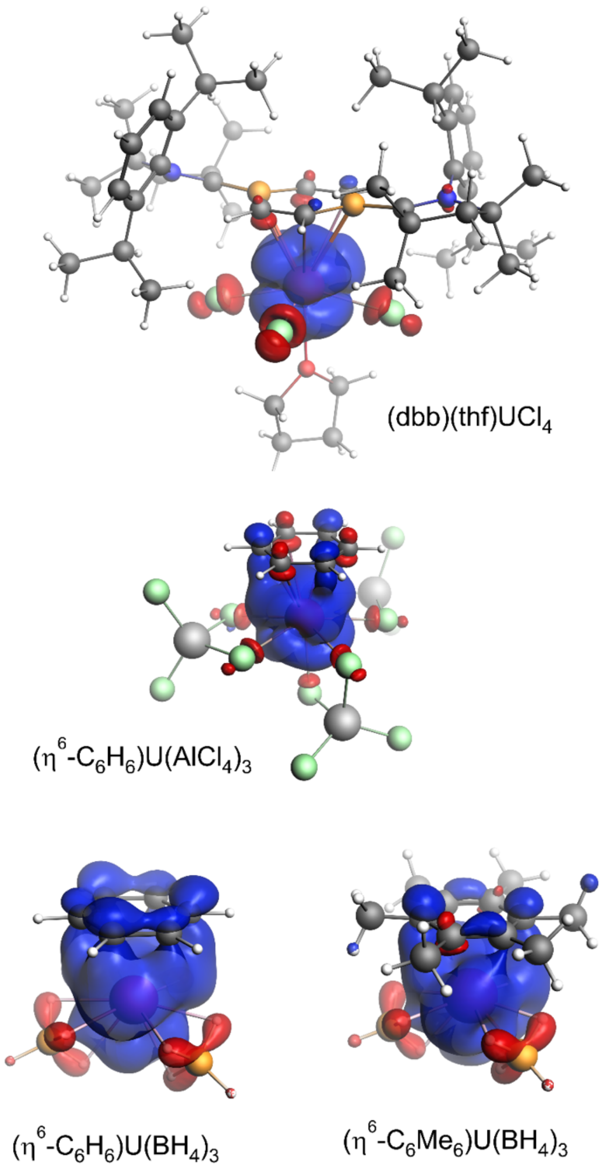

Figure S28. Spin-density distributions in **3a**, [(η^6-C_6H_6)U(BH_4)$_3$], [(η^6-C_6Me_6)U(BH_4)$_3$], and [(η^6-C_6H_6)U($AlCl_4$)$_3$] (isosurface plots ± 0.001 a.u.; a blue surface indicates positive spin density and a red one indicates negative spin density).

Table S2. Selected X-ray and DFT optimized interatomic distances (in pm) of **1**, **2a**, and **3a**.[a,b]

	**1**		**2a**		**3a**	
	DFT	X-ray	DFT	X-ray	DFT	X-ray
d(M···C_{dbb})	–	–	295.3	293.8	288.3	288.3
d(M···B)	–	–	304.6	304.9	298.0	299.1
d(C_{dbb}–C_{dbb})	137.7	137.5	139.4	140.1	139.4	139.8
d(B–C_{dbb})	152.2	153.2	151.5	151.7	151.4	151.1
d(B–C_{cAAC})	155.3	155.9	157.6	158.4	157.5	158.9
d(N–C_{cAAC})	133.5	134.1	131.5	131.6	131.5	131.8

[a] Only the averaged values are given for chemically equivalent bonds. [b] Optimized at the PBE0-D3(BJ)/def2-TZVP/ECP level.

Table S3. QTAIM delocalization indices (DI), as a measure of the bond-covalency, for selected atom pairs in compounds **1**, **2a**, and **3a**. Partial charges of the central aromatic unit, *q*(Ar) and oxidation state of the actinide center according to LOBA analysis.[a]

	**1**	**2a**	**3a**
DI (M···C_{dbb})	-	0.174	0.200
DI (M···B)	-	0.050	0.055
DI (C_{dbb}–C_{dbb})	1.603	1.493	1.482
DI (B–C_{dbb})	0.610	0.621	0.622
DI (B–C_{cAAC})	0.540	0.501	0.500
DI (N–C_{cAAC})	1.214	1.289	1.287
q(dbb)	–0.576	–0.289	–0.260
LOBA	-	Th(IV)	U(IV)

[a] PBE0-D3(BJ)/def2-TZVP/ECP results.

Table S4. Selected ^{1}H, ^{13}C, and ^{11}B NMR shifts (in ppm) of free diborabenzene (dbb) ligand (**1**), **2a,** and [(dbb)W(CO)$_3$].[a]

	**1**		**2a**		[(dbb)W(CO)$_3$]	
	calcd.	*expt.*	*calcd.*	*expt.*	*calcd.*	*expt.*
$\delta(^{13}C_{dbb})$	156.7	150.5	158.3	150.6	115.1	109.3
$\delta(^{11}B)$	22.6	24.8	25.7	27.8	2.8	7.0
$\delta(^{1}H_{dbb})$	7.15	7.31	7.72	7.18	4.35	4.78

[a] 2c-ZORA-SO/PBE0/TZ2P results for PBE0-D3(BJ)/def2-TZVP/ECP structures (*cf.* Computational Details)

Table S5. Results obtained from energy decomposition analysis (EDA, in kJ/mol) for [(dbb)(thf)AnCl$_4$] and related, hypothetical [(η^6-C_6H_6)(thf)AnCl$_4$] complexes.[a]

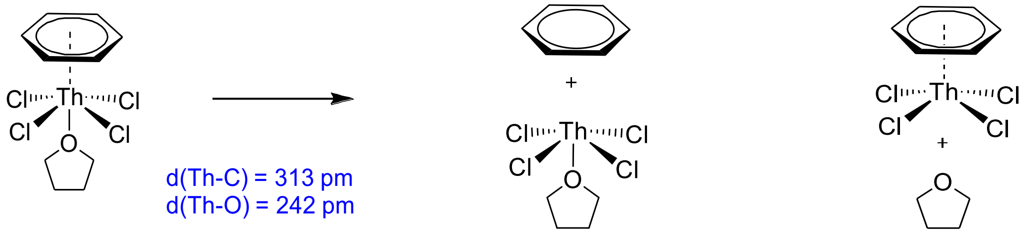

complex	fragments	
Pauli repulsion	179.3	248.9
Electrostatic interactions	-130.6	-241.9
Orbital interactions	-143.1	-136.2
Total bonding energy	**-94.4**	**-129.2**

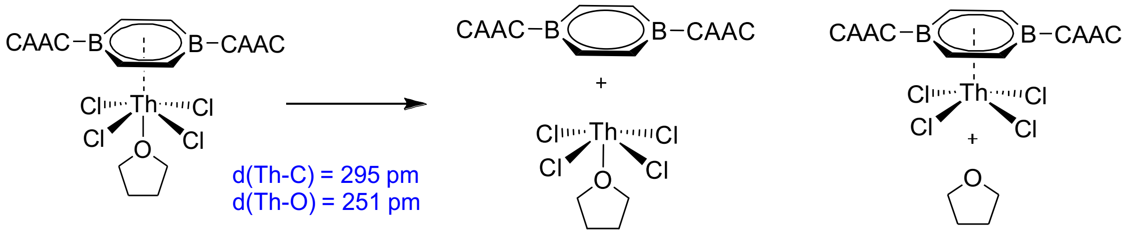

complex	fragments	
Pauli repulsion	457.0	229.3
Electrostatic interactions	-388.2	-207.5
Orbital interactions	-345.7	-107.6
Total bonding energy	**-276.9**	**-85.8**

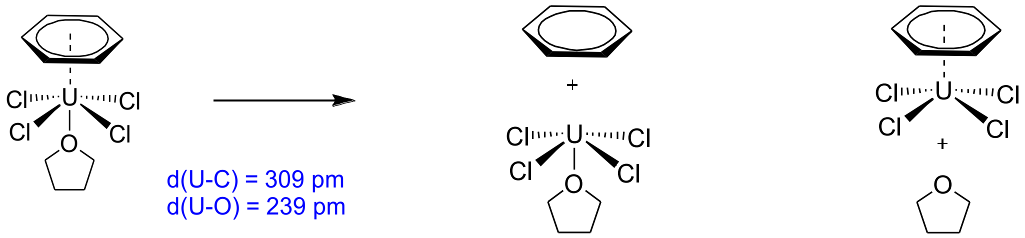

complex	fragments	
Pauli repulsion	205.3	256.9
Electrostatic interactions	-145.9	-244.7
Orbital interactions	-164.6	-172.0
Total bonding energy	**-105.2**	**-159.8**

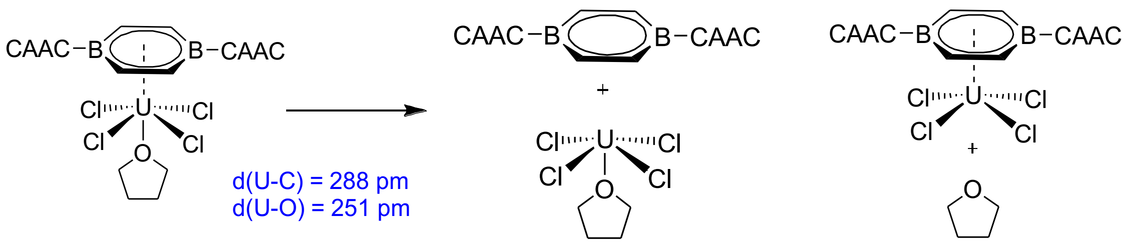

complex	fragments	
Pauli repulsion	525.8	232.3
Electrostatic interactions	-416.5	-206.5
Orbital interactions	-392.5	-113.6
Total bonding energy	**-283.2**	**-87.8**

[a] 2c-ZORA-SR/PBE0/def2-TZVP results for PBE0-D3(BJ)/def2-TZVP/ECP optimized structures (*cf.* Computational details). The An-L distances are averaged over chemically equivalent bonds.

Table S6. Results obtained from energy decomposition analysis (EDA, in kJ/mol) for other literature-known actinide complexes with non-tethered arene ligands.[a]

complex	fragments

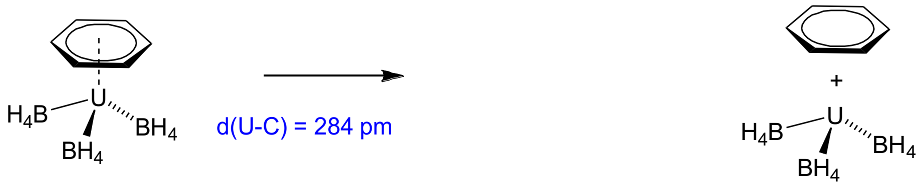

Pauli repulsion	387.0
Electrostatic interactions	-257.3
Orbital interactions	-303.9
Total bonding energy	**-174.2**

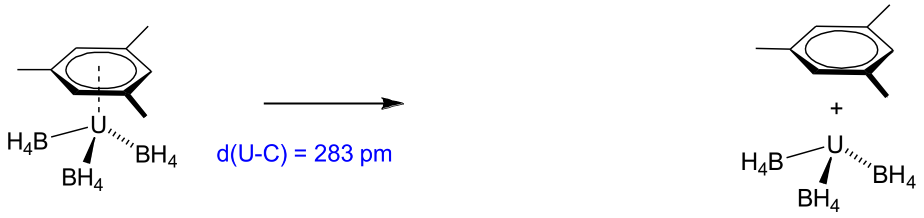

Pauli repulsion	431.9
Electrostatic interactions	-309.6
Orbital interactions	-305.2
Total bonding energy	**-182.9**

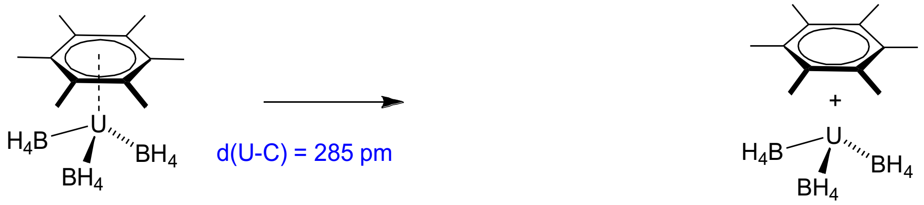

Pauli repulsion	422.6
Electrostatic interactions	-322.4
Orbital interactions	-299.6
Total bonding energy	**-199.4**

complex	fragments
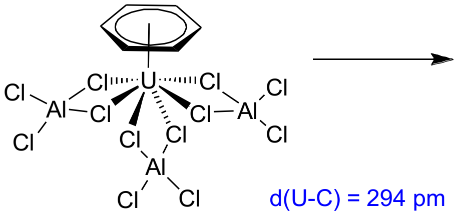 d(U-C) = 294 pm	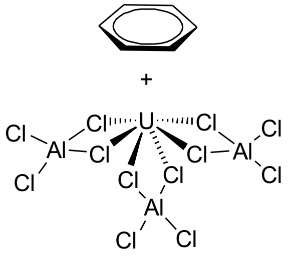
Pauli repulsion	316.2
Electrostatic interactions	-229.9
Orbital interactions	-263.2
Total bonding energy	**-176.9**

[a] 2c-ZORA-SR/PBE0/def2-TZVP results for PBE0-D3(BJ)/def2-TZVP/ECP optimized structures (*cf.* Computational details). The An-L distances are averaged over chemically equivalent bonds.

Cartesian coordinates and SCF energies of DFT optimized structures:

Diborabenzene **1** (dbb): E_{SCF} = -1874.162623164 a.u.

B	1.03866	-0.85210	-1.47723
C	0.01731	-0.57499	-0.38171
H	-0.78089	-1.26293	-0.12347
B	1.07161	1.67640	0.13683
C	0.02880	0.58849	0.35406
H	-0.75538	0.65838	1.10082
C	2.08032	0.23546	-1.69579
H	2.87571	0.15070	-2.43413
C	2.09855	1.39525	-0.95105
H	2.88727	2.09738	-1.21459
C	1.07131	-2.14497	-2.33754
N	0.33201	-3.24316	-2.17071
C	1.98825	-2.37785	-3.53354
C	3.44080	-2.54499	-3.07528
H	4.07473	-2.76085	-3.94048
H	3.54925	-3.36730	-2.36578
H	3.80696	-1.64361	-2.58780
C	1.88424	-1.26097	-4.57221
H	2.42313	-1.56223	-5.47652
H	2.29543	-0.32004	-4.21722
H	0.83989	-1.08630	-4.84186
C	1.45145	-3.67313	-4.15921
H	0.78660	-3.42333	-4.99008
H	2.25065	-4.30233	-4.55538
C	0.64960	-4.38471	-3.08088
C	1.46288	-5.45495	-2.36149
H	1.70789	-6.24595	-3.07327
H	0.88727	-5.90206	-1.55105
H	2.39506	-5.06591	-1.95476
C	-0.59838	-5.04581	-3.64126
H	-0.29367	-5.87468	-4.28325
H	-1.18622	-4.35556	-4.24333
H	-1.23062	-5.44873	-2.84770
C	-0.71039	-3.38034	-1.20127
C	-0.42067	-3.85037	0.08589
C	0.98423	-4.09584	0.58672
H	1.66459	-3.98512	-0.25697
C	1.15606	-5.49306	1.17571
H	2.20796	-5.67565	1.40864

H	0.81636	-6.27923	0.49865
H	0.59463	-5.59812	2.10745
C	1.38009	-3.04266	1.62039
H	2.40233	-3.22192	1.96430
H	0.72065	-3.08580	2.49177
H	1.32385	-2.03931	1.19813
C	-1.47756	-4.00582	0.97675
H	-1.27260	-4.36478	1.97878
C	-2.77201	-3.68417	0.61962
H	-3.58016	-3.80909	1.33127
C	-3.02881	-3.17294	-0.63878
H	-4.03862	-2.87975	-0.90123
C	-2.01111	-3.00097	-1.56794
C	-2.33855	-2.32389	-2.88088
H	-1.45708	-2.38159	-3.52114
C	-3.50284	-2.99033	-3.60725
H	-3.63508	-2.54103	-4.59482
H	-4.44016	-2.85333	-3.06282
H	-3.34976	-4.06348	-3.73575
C	-2.63335	-0.84094	-2.65867
H	-2.84090	-0.35082	-3.61385
H	-1.79022	-0.33886	-2.18441
H	-3.50987	-0.71291	-2.01724
C	1.12014	3.01242	0.92775
N	0.22051	3.46682	1.80253
C	2.22490	4.05690	0.81108
C	3.61435	3.46277	1.04192
H	4.34211	4.27708	1.12114
H	3.92208	2.79545	0.24202
H	3.63778	2.89538	1.97530
C	2.16300	4.75348	-0.55209
H	2.93491	5.52730	-0.60386
H	1.19591	5.23210	-0.71660
H	2.31888	4.04594	-1.36396
C	1.92338	5.03981	1.95168
H	2.55092	4.79656	2.81297
H	2.13562	6.07325	1.67191
C	0.46352	4.84348	2.32922
C	0.24200	4.93912	3.82920
H	0.42103	5.97013	4.14098
H	0.92621	4.29822	4.38194
H	-0.78267	4.67775	4.10055
C	-0.45079	5.86071	1.65642

H	-0.20836	6.85532	2.03643
H	-1.49593	5.65982	1.88990
H	-0.33182	5.87513	0.57382
C	-0.91800	2.72902	2.25539
C	-2.13848	2.81915	1.57289
C	-2.30835	3.55235	0.26183
H	-1.38182	4.08688	0.05426
C	-3.45635	4.55711	0.30327
H	-3.46525	5.15574	-0.61078
H	-3.38859	5.23670	1.15503
H	-4.42175	4.04847	0.36715
C	-2.52031	2.56544	-0.88495
H	-2.62422	3.10560	-1.82973
H	-3.43114	1.98032	-0.73078
H	-1.67981	1.87614	-0.96788
C	-3.22528	2.11656	2.08330
H	-4.17601	2.17004	1.56563
C	-3.10866	1.33243	3.21420
H	-3.96816	0.79141	3.59358
C	-1.88295	1.21599	3.84258
H	-1.78309	0.56475	4.70302
C	-0.76613	1.89792	3.37657
C	0.56961	1.63326	4.03676
H	1.29645	2.33879	3.62998
C	1.06581	0.23002	3.69008
H	2.04328	0.05101	4.14629
H	1.15565	0.10002	2.61143
H	0.37347	-0.52824	4.06671
C	0.52014	1.81993	5.55019
H	1.52388	1.72698	5.97232
H	-0.10111	1.05616	6.02395
H	0.11803	2.79461	5.83325

Complex **2a**: E_{SCF} = -4122.499132546 a.u.

Th	-1.28984	2.02956	0.49868
Cl	-3.41479	1.00931	1.75158
Cl	-0.35226	2.64826	2.94432
Cl	0.28467	3.93647	-0.49547
Cl	-2.87476	2.32146	-1.66193
O	-2.58517	4.10389	1.05392
C	-1.99423	5.35973	1.45741

H	-1.20969	5.13544	2.17884
H	-1.54765	5.81579	0.57185
C	-3.14545	6.17014	2.00845
H	-3.32160	5.92030	3.05809
H	-2.95908	7.24219	1.93557
C	-4.30605	5.69335	1.14630
H	-5.28494	5.89128	1.58438
H	-4.26715	6.16364	0.16026
C	-4.02672	4.21134	1.03884
H	-4.40984	3.65020	1.89345
H	-4.37887	3.74680	0.11887
B	-0.37918	-0.02175	-1.54204
C	-1.21203	-0.72273	-0.49364
C	-0.79951	-0.83194	0.83348
B	0.52798	-0.28648	1.32770
C	1.29347	0.52891	0.30714
C	0.86776	0.66088	-1.01400
H	-2.18487	-1.14712	-0.71491
H	-1.52103	-1.30400	1.49010
H	2.20014	1.07007	0.55799
H	1.49491	1.29539	-1.63227
C	-0.75100	0.03600	-3.07205
N	-1.58111	-0.77392	-3.68886
C	-1.68665	-0.56106	-5.17800
C	-0.97605	0.77423	-5.32990
C	-0.09320	0.94912	-4.09370
C	-2.18669	-1.94821	-3.10652
C	-3.54599	-1.95245	-2.75009
C	-4.08568	-3.14671	-2.28257
C	-3.32150	-4.28633	-2.13933
C	-1.97609	-4.24364	-2.44572
C	-1.37932	-3.08636	-2.92978
H	-5.13096	-3.16678	-1.99953
H	-3.76922	-5.20086	-1.76743
H	-1.36420	-5.12553	-2.29836
C	-4.44768	-0.73493	-2.72845
H	-3.87672	0.12890	-3.07150
C	-4.89850	-0.42441	-1.30185
H	-4.06665	-0.26915	-0.61986
H	-5.52483	-1.22799	-0.90541
H	-5.48362	0.49666	-1.29532
C	-5.69444	-0.90611	-3.59540
H	-5.47349	-1.17366	-4.62786

H	-6.26517	0.02531	-3.60056
H	-6.34600	-1.68275	-3.18612
C	0.11446	-3.12424	-3.17131
H	0.42303	-2.16802	-3.59206
C	0.86549	-3.28595	-1.85344
H	0.62215	-4.23961	-1.37653
H	1.94319	-3.26378	-2.03086
H	0.61280	-2.48506	-1.16007
C	0.52243	-4.22823	-4.14269
H	1.59529	-4.17045	-4.34309
H	0.32451	-5.21617	-3.72058
H	-0.00624	-4.16336	-5.09415
C	-1.01507	-1.69996	-5.93197
H	0.04207	-1.80305	-5.69657
H	-1.10526	-1.50497	-7.00182
H	-1.51403	-2.64728	-5.72126
C	-3.12236	-0.47810	-5.65036
H	-3.11382	-0.25033	-6.71833
H	-3.64017	-1.42792	-5.51634
H	-3.67169	0.31061	-5.13986
H	-0.40270	0.82546	-6.25634
H	-1.71770	1.57609	-5.34867
C	1.33213	0.43212	-4.35138
H	1.93292	0.45127	-3.44423
H	1.80493	1.08011	-5.09354
H	1.34735	-0.58682	-4.73998
C	-0.03219	2.42134	-3.71121
H	0.60386	2.61626	-2.85206
H	0.36488	2.98083	-4.56333
H	-1.02316	2.80436	-3.47101
C	0.99680	-0.51677	2.81581
N	2.14904	-0.15457	3.34180
C	2.22592	-0.33927	4.83772
C	1.17294	-1.41503	5.03002
C	0.18559	-1.27194	3.86277
C	3.33302	0.15811	2.58029
C	3.99496	-0.92842	1.98192
C	5.12719	-0.67063	1.22108
C	5.61668	0.61239	1.07501
C	4.97589	1.65885	1.70488
C	3.82714	1.46317	2.46592
H	5.63479	-1.49623	0.73620
H	6.50145	0.79470	0.47607

H	5.36092	2.66546	1.59522
C	3.54541	-2.36681	2.11359
H	2.75525	-2.41137	2.86440
C	4.67194	-3.28051	2.58921
H	4.28107	-4.28178	2.78547
H	5.45473	-3.37959	1.83385
H	5.14196	-2.91106	3.50281
C	2.96213	-2.87630	0.80143
H	2.59783	-3.90080	0.91259
H	3.72204	-2.87230	0.01507
H	2.13536	-2.24916	0.47059
C	3.19115	2.67361	3.10185
H	2.24367	2.37016	3.54384
C	4.10299	3.24610	4.18678
H	4.42116	2.49359	4.91210
H	5.00857	3.66796	3.74276
H	3.59204	4.04797	4.72515
C	2.86540	3.75735	2.08126
H	3.77062	4.17559	1.63280
H	2.32739	4.57114	2.57137
H	2.22255	3.38882	1.28409
C	3.60479	-0.77980	5.28457
H	3.90443	-1.71725	4.81625
H	4.36158	-0.02462	5.06557
H	3.58353	-0.93310	6.36517
C	1.82983	0.94220	5.56457
H	2.61905	1.68919	5.53418
H	1.64762	0.69753	6.61328
H	0.92488	1.38017	5.14503
H	0.67781	-1.33023	5.99778
H	1.65039	-2.39803	4.98574
C	-1.09715	-0.54882	4.27119
H	-0.89977	0.43289	4.69805
H	-1.61643	-1.15647	5.01689
H	-1.76878	-0.39306	3.42791
C	-0.16146	-2.68299	3.36794
H	0.70863	-3.18690	2.94140
H	-0.49866	-3.26708	4.22918
H	-0.95246	-2.69096	2.62380

Complex **3a**: E_{SCF} = -4424.008000847 a.u.

U	-1.27425	-1.93816	0.60903
Cl	-3.27593	-0.78941	1.81029
Cl	-2.76196	-2.18564	-1.54384
Cl	0.11441	-3.92098	-0.33028
Cl	-0.38206	-2.62705	2.97862
O	-2.78664	-3.86072	1.16900
C	-4.22694	-3.85258	1.10586
H	-4.51277	-3.38362	0.16501
H	-4.59229	-3.24098	1.93351
C	-4.63696	-5.30354	1.23451
H	-4.62104	-5.79516	0.25828
H	-5.63719	-5.40784	1.65647
C	-3.53735	-5.86244	2.12639
H	-3.44046	-6.94743	2.07392
H	-3.71019	-5.58037	3.16849
C	-2.31404	-5.15768	1.58455
H	-1.52040	-5.00137	2.31367
H	-1.89878	-5.66180	0.70873
B	0.56386	0.21837	1.47520
C	-0.68141	0.90915	0.96102
C	-1.08673	0.83653	-0.37026
B	-0.30885	0.05734	-1.40876
C	0.84336	-0.76018	-0.85656
C	1.25072	-0.68176	0.47464
H	-1.33602	1.48721	1.60552
H	-2.00285	1.36736	-0.60742
H	1.39081	-1.48300	-1.45073
H	2.07415	-1.33203	0.74868
C	1.06743	0.44122	2.94933
N	2.28109	0.19230	3.38917
C	2.52957	0.58841	4.82300
C	1.10936	0.84470	5.29947
C	0.26604	1.11214	4.05339
C	3.40823	-0.18591	2.57034
C	4.01437	0.80893	1.78191
C	5.15970	0.47380	1.07116
C	5.69628	-0.79667	1.12620
C	5.06036	-1.76827	1.87062
C	3.90190	-1.50128	2.59341
H	5.63288	1.22900	0.45462
H	6.59557	-1.03552	0.57012

H	5.45412	-2.77745	1.87699
C	3.47085	2.20925	1.59892
H	2.60415	2.33489	2.24710
C	2.98219	2.40625	0.16698
H	2.23081	1.66293	-0.09538
H	2.54154	3.39974	0.05472
H	3.80867	2.32302	-0.54445
C	4.49259	3.28667	1.95212
H	4.03273	4.27491	1.87271
H	5.33985	3.26675	1.26261
H	4.88647	3.17407	2.96275
C	3.21680	-2.68119	3.25281
H	2.30993	-2.32854	3.74546
C	2.77982	-3.70109	2.20236
H	2.11815	-3.27643	1.45209
H	3.64586	-4.13373	1.69504
H	2.22842	-4.51060	2.68400
C	4.10901	-3.39494	4.26787
H	4.51322	-2.73232	5.03174
H	4.95333	-3.87712	3.76875
H	3.53770	-4.18089	4.76716
C	3.43073	1.81315	4.89212
H	3.01544	2.67812	4.38040
H	4.41075	1.59314	4.46499
H	3.57412	2.07683	5.94123
C	3.18699	-0.51384	5.62552
H	2.60483	-1.43283	5.60475
H	4.19815	-0.71518	5.27095
H	3.25718	-0.17949	6.66273
H	1.06178	1.66768	6.01438
H	0.73039	-0.05268	5.79376
C	0.18746	2.61701	3.74632
H	-0.31680	2.80250	2.79977
H	-0.38176	3.10277	4.54287
H	1.16818	3.09129	3.70071
C	-1.14144	0.57099	4.26351
H	-1.13353	-0.51145	4.38563
H	-1.81366	0.79573	3.43989
H	-1.55080	1.02515	5.17064
C	-0.67004	0.00841	-2.94399
N	-1.60539	0.70231	-3.55819
C	-1.83433	0.28829	-4.99101
C	-0.49000	-0.34015	-5.30877

C	0.08786	-0.82547	-3.97149
C	-2.21352	1.90313	-3.03925
C	-1.42252	3.06543	-3.06939
C	-1.96230	4.24172	-2.56673
C	-3.25234	4.28661	-2.07516
C	-4.02467	3.14388	-2.09133
C	-3.53213	1.93278	-2.56908
H	-1.35676	5.14040	-2.56411
H	-3.65661	5.21403	-1.68609
H	-5.03750	3.17716	-1.70892
C	-0.00991	3.10987	-3.60978
H	0.19035	2.16767	-4.12143
C	0.18378	4.22723	-4.63199
H	-0.55845	4.18712	-5.43235
H	0.11368	5.21343	-4.16763
H	1.17647	4.15043	-5.08231
C	0.99983	3.24170	-2.47644
H	0.89071	2.42902	-1.75947
H	2.02137	3.22790	-2.86534
H	0.85501	4.18332	-1.93995
C	-4.45288	0.73899	-2.53113
H	-3.87972	-0.14207	-2.81488
C	-4.99090	0.47478	-1.13010
H	-5.55639	-0.45913	-1.12469
H	-5.66054	1.27347	-0.79935
H	-4.19218	0.36785	-0.39877
C	-5.61751	0.92956	-3.50223
H	-6.21957	0.01897	-3.55369
H	-6.26893	1.74018	-3.16487
H	-5.28950	1.18164	-4.51348
C	-2.15495	1.47172	-5.88009
H	-2.27752	1.11110	-6.90310
H	-1.35501	2.21206	-5.87693
H	-3.08297	1.96202	-5.58151
C	-2.95265	-0.74522	-5.08159
H	-3.93424	-0.29288	-4.96501
H	-2.91517	-1.20456	-6.07150
H	-2.84275	-1.52638	-4.32961
H	-0.58119	-1.15045	-6.03283
H	0.16937	0.41893	-5.73915
C	1.58268	-0.47697	-3.95071
H	2.03545	-0.88835	-4.85726
H	1.74462	0.60349	-3.95495

H	2.10356	-0.88995	-3.09205
C	-0.08701	-2.33141	-3.77827
H	-1.12383	-2.64179	-3.89300
H	0.51660	-2.85066	-4.52747
H	0.23137	-2.65932	-2.79036

$[(\eta^6\text{-}C_6H_6)(thf)ThCl_4]$: E_{SCF} = -2712.640623130 a.u.

Th	0.32390	-0.51266	-0.10093
Cl	1.49559	-0.11488	2.21885
Cl	-1.63381	-1.74077	1.14876
Cl	1.99807	1.21020	-1.17266
Cl	-1.17872	-0.44043	-2.26279
C	1.01267	-3.55468	0.09925
C	0.56154	-3.42937	-1.21246
C	1.30020	-2.69534	-2.13779
C	2.48463	-2.07478	-1.74784
C	2.93037	-2.19072	-0.43306
C	2.19858	-2.93538	0.48905
H	-0.38465	-3.86790	-1.50347
H	0.92899	-2.57168	-3.14647
H	3.03629	-1.46612	-2.45209
H	3.82461	-1.66742	-0.11871
H	2.52876	-2.99596	1.51744
H	0.41985	-4.09594	0.82462
O	-0.91828	1.48015	0.47320
C	-0.32625	2.76635	0.80677
C	-1.48386	3.60505	1.29974
C	-2.65438	3.04942	0.49829
C	-2.36855	1.56658	0.50425
H	-2.70738	1.07324	1.41731
H	-2.74622	1.02537	-0.36196
H	-2.64024	3.43553	-0.52396
H	-3.62551	3.27552	0.93876
H	-1.30889	4.66714	1.12813
H	-1.64408	3.45157	2.36983
H	0.45401	2.58537	1.54505
H	0.12278	3.16253	-0.10511

[(η^6-C_6H_6)(thf)UCl_4]: E_{SCF} = -2781.800468809 a.u.

U	0.31801	-0.51373	-0.13706
Cl	1.42869	-0.11243	2.14560
Cl	-1.61237	-1.69831	1.06836
Cl	1.95358	1.16641	-1.18501
Cl	-1.11855	-0.44363	-2.26877
C	1.03515	-3.51151	0.16681
C	0.54581	-3.42974	-1.13395
C	1.24679	-2.71220	-2.09885
C	2.43421	-2.06888	-1.76067
C	2.92103	-2.14672	-0.45893
C	2.22405	-2.87097	0.50411
H	-0.39876	-3.89484	-1.38651
H	0.85025	-2.62578	-3.10173
H	2.96159	-1.48066	-2.50004
H	3.82376	-1.61400	-0.18756
H	2.58920	-2.90758	1.52182
H	0.47370	-4.04564	0.92191
O	-0.90895	1.46643	0.41912
C	-0.31575	2.72598	0.83767
C	-1.47423	3.54088	1.36742
C	-2.62848	3.06486	0.49482
C	-2.35773	1.58101	0.39018
H	-2.74791	1.02253	1.24332
H	-2.70378	1.11867	-0.53305
H	-2.58366	3.53406	-0.49117
H	-3.60932	3.26650	0.92641
H	-1.28387	4.61158	1.28741
H	-1.66303	3.30298	2.41733
H	0.45407	2.49699	1.57302
H	0.14649	3.17772	-0.04198

[(η^6-C_6H_6)$U(BH_4)_3$]: E_{SCF} = -790.6528471656 a.u.

U	-0.21925	-0.31534	-0.10727
B	-1.93366	0.21525	-1.85862
B	-1.46997	-1.11810	1.91348
B	1.09999	-2.27320	-0.95454
H	-0.75824	0.36517	-2.25637
H	-2.02178	0.94226	-0.84457
H	-2.74839	0.47404	-2.69331

H	-1.99911	-0.95383	-1.43904
H	-0.22830	-1.15809	2.05426
H	-1.71028	-1.77677	0.88533
H	-1.72156	0.06982	1.61830
H	-2.06539	-1.49920	2.87661
H	1.23133	-1.24712	-1.65830
H	-0.12483	-2.48586	-0.89925
H	1.73152	-3.20282	-1.35971
H	1.43174	-1.91621	0.19608
C	0.08463	2.43071	0.56489
C	0.68669	2.31218	-0.69297
C	1.82178	1.51699	-0.85470
C	2.35246	0.82864	0.24242
C	1.75515	0.94495	1.49854
C	0.61751	1.74474	1.65699
H	-0.80008	3.04214	0.68652
H	0.26287	2.82937	-1.54395
H	2.28264	1.41947	-1.82932
H	3.22029	0.19503	0.11296
H	2.16158	0.40722	2.34561
H	0.14249	1.81896	2.62668

$[(\eta^6\text{-}C_6H_3Me_3)U(BH_4)_3]$: E_{SCF} = -908.5470496529 a.u.

U	-0.25114	-0.35797	-0.11776
B	-1.76083	0.09456	-2.07290
B	-1.71204	-0.94843	1.83701
B	1.11077	-2.39188	-0.68237
H	-0.54730	0.18103	-2.35507
H	-1.92736	0.89000	-1.12367
H	-2.47452	0.32387	-3.00392
H	-1.91397	-1.04073	-1.59090
H	-0.49170	-1.00967	2.09675
H	-1.88426	-1.69165	0.85525
H	-1.89329	0.21339	1.41813
H	-2.40297	-1.22261	2.77303
H	1.30151	-1.43606	-1.46408
H	-0.11459	-2.60206	-0.70196
H	1.76750	-3.35340	-0.95207
H	1.35639	-1.93404	0.45281
C	0.03166	2.36324	0.62316
C	0.59212	2.27190	-0.65720

C	1.73672	1.50585	-0.91014
C	2.30078	0.79063	0.15344
C	1.77186	0.85254	1.44922
C	0.62809	1.63326	1.65898
C	-1.16147	3.22640	0.88025
C	2.34708	1.46156	-2.27413
C	2.41741	0.11527	2.57818
H	0.12199	2.80229	-1.47714
H	3.16473	0.16332	-0.03304
H	0.18621	1.66485	2.64807
H	-1.79099	3.30276	-0.00676
H	-1.76514	2.83103	1.69778
H	-0.84359	4.23682	1.15537
H	2.83551	0.50371	-2.45600
H	1.59519	1.61723	-3.04805
H	3.10169	2.24783	-2.37483
H	1.68514	-0.16181	3.33681
H	2.90831	-0.79225	2.22585
H	3.17529	0.74417	3.05541

[(η^6-C_6Me_6)$U(BH_4)_3$]: E_{SCF} = -1026.394460580 a.u.

U	-0.25852	-0.36930	-0.12201
B	-1.82768	0.13326	-2.02566
B	-1.65529	-1.01271	1.87224
B	1.11618	-2.37919	-0.76621
H	-0.62401	0.22888	-2.34093
H	-1.96769	0.90241	-1.05173
H	-2.56689	0.39166	-2.93034
H	-1.97135	-1.01410	-1.57290
H	-0.42746	-1.06299	2.09273
H	-1.85025	-1.74595	0.88880
H	-1.86740	0.15158	1.47864
H	-2.31095	-1.30823	2.82857
H	1.28600	-1.40095	-1.52420
H	-0.10544	-2.60467	-0.77687
H	1.78217	-3.32436	-1.07376
H	1.37504	-1.95306	0.37679
C	0.03245	2.37062	0.58972
C	0.62470	2.29434	-0.68932
C	1.75826	1.48498	-0.89533
C	2.33670	0.79603	0.19194

C	1.74602	0.86745	1.46824
C	0.61244	1.68325	1.67264
C	-1.18693	3.22174	0.79375
C	0.08310	3.15051	-1.79630
C	2.38271	1.38041	-2.25633
C	3.62728	0.05792	-0.01320
C	2.33871	0.11585	2.62436
C	0.08729	1.86825	3.06649
H	-1.73313	3.36011	-0.13617
H	-1.87412	2.76659	1.50558
H	-0.91816	4.21311	1.17478
H	0.76352	3.19515	-2.64214
H	-0.87836	2.78274	-2.16014
H	-0.06482	4.17169	-1.43982
H	2.96418	0.46713	-2.35585
H	1.62638	1.36654	-3.03994
H	3.05136	2.22588	-2.45105
H	4.09394	-0.20764	0.93126
H	3.48303	-0.86201	-0.58396
H	4.33192	0.68335	-0.56448
H	1.60181	-0.05258	3.40578
H	2.70706	-0.86072	2.31412
H	3.17685	0.66533	3.06669
H	-0.59923	2.70810	3.13016
H	-0.43772	0.97974	3.42382
H	0.91193	2.06562	3.75384

[(η^6-C_6H_6)U($AlCl_4$)$_3$]: E_{SCF} = -6957.776410908 a.u.

U	-0.30990	-0.39702	-0.06195
Al	2.29719	-3.17726	-0.27311
Al	-2.93017	1.34367	-2.21227
Al	-2.67249	-1.98937	2.34582
Cl	4.27284	-2.43822	-0.28230
Cl	-1.22039	-0.31385	2.63272
Cl	-2.07764	-3.61460	3.51084
Cl	-4.61335	-1.24039	2.50825
Cl	1.15331	-2.09407	-1.84015
Cl	2.00819	-5.23306	-0.43018
Cl	1.22770	-2.31519	1.45266
Cl	-1.29399	-0.07172	-2.71923
Cl	-2.32426	3.28032	-2.78813

Cl	-4.76119	0.62999	-2.89859
Cl	-2.73044	1.18066	-0.02308
Cl	-2.21424	-2.41830	0.20187
C	1.71505	1.34344	-1.25199
C	2.41187	0.71647	-0.21949
C	2.04620	0.94728	1.10577
C	0.98991	1.80959	1.39973
C	0.30049	2.44347	0.36650
C	0.66145	2.20950	-0.96097
H	1.98409	1.14663	-2.28243
H	3.22100	0.03098	-0.44224
H	2.57429	0.44217	1.90419
H	0.70178	1.98230	2.42866
H	-0.52562	3.10632	0.58808
H	0.10831	2.69023	-1.75899

S6 References

[1] M. Arrowsmith, J. Böhnke, H. Braunschweig, M. A. Celik, C. Claes, W. C. Ewing, I. Krummenacher, K. Lubitz, C. Schneider, *Angew. Chem. Int. Ed.* **2016**, *55*, 11271.

[2] T. Cantat, B. L. Scott, J. L. Kiplinger, *Chem. Commun.* **2010**, *46*, 919.

[3] J. L. Kiplinger, D. E. Morris, B. L. Scott, C. J., Burns, *Organometallics* **2002**, *21*, 5978.

[4] G. Sheldrick, *Acta Cryst.* **2015**, *A71*, 3.

[5] G. Sheldrick, *Acta Cryst.* **2008**, *A64*, 112.

[6] A. L. Spek, *Acta Cryst.* **2015**, *C71*, 9.

[7] I. Castro-Rodriguez, H. Nakai, L. N. Zakharov, A. L. Rheingold, K. Meyer, *Science* **2004**, *305*, 1757.

[8] J. Jung, M. Atanasov, F. Neese, *Inorg. Chem.* **2017**, *56*, 8802.

[9] D. R. Kindra, W. J. Evans, *Chem. Rev.* **2014**, *114*, 8865.

[10] a) J. P. Perdew, K. Burke, M. Ernzerhof, *Phys. Rev. Lett.* **1996**, *77*, 3865; b) J. P. Perdew, K. Burke, M. Ernzerhof, *Phys. Rev. Lett.* **1997**, *78*, 1396; c) S. Adamo, V. Barone, *Chem. Phys. Lett.* **1998**, *298*, 113.

[11] S. Grimme, J. Antony, S. Ehrlich, H. Krieg, *J. Chem. Phys.* **2010**, *132*, 154104.

[12] S. Grimme, S. Ehrlich, L. Goerigk, *J. Comp. Chem.* **2011**, *32*, 1456.

[13] Turbomole, version 7.0.2, A Development of University of Karlsruhe and Forschungszentrum Karlsruhe GmbH, 1989-2007; TURBOMOLE GmbH, since **2007**; available from http://www.turbomole.com.

[14] X. Cao, M. Dolg, *J. Mol. Struct. THEOCHEM* **2004**, *673*, 203.

[15] F. Weigend, R. Ahlrichs, *Phys. Chem. Chem. Phys.* **2005**, *7*, 3297.

[16] Amsterdam Density Functional (ADF), version 2017.112, SCM, Theoretical Chemistry, Vrije Universiteit, Amsterdam, Netherlands, **2018**; available from http://www.scm.com.

[17] a) G. Schreckenbach, T. Ziegler, *J. Phys. Chem.* **1995**, *99*, 606; b) S. K. Wolff, T. Ziegler, *J. Chem. Phys.* **1998**, *109*, 895; c) S. K. Wolff, T. Ziegler, E. van Lenthe, E. J. Baerends, *J. Chem. Phys.* **1999**, *110*, 7689; d) M. Krykunov, T. Ziegler, E. van Lenthe, *Int. J. Quantum Chem.* **2009**, *109*, 1676.

[18] J. Autschbach, *Mol. Phys.* **2013**, *111*, 2544.

[19] A. H. Greif, P. Hrobárik, J. Autschbach, M. Kaupp, *Phys. Chem. Chem. Phys.* **2016**, *18*, 30462.

[20] J. R. Cheeseman, G. W. Trucks, T. A. Keith, M. J. Frisch, *J. Chem. Phys.* **1996**, *104*, 5497.

[21] A. E. Reed, L. A. Curtiss, F. Weinhold, *Chem. Rev.* **1998**, *88*, 899.

[22] a) R. F. W. Bader, M. E. Stephens, *J. Am. Chem. Soc.* **1975**, *97*, 7391; b) R. F. W. Bader, *Atoms in Molecules: A Quantum Theory*, Oxford University Press, Oxford, UK, **1990**; c) C. F. Matta, R. J. Boyd, *The Quantum Theory of Atoms in Molecules*, Wiley-VCH, Weinheim, Germany, **2007**.

[23] Gaussian 09, revision D.01, Frisch, M. J. et al. Gaussian, Inc., Wallingford CT, **2009**.

[24] T. Lu, Multiwfn: A Multifunctional Wave Function Analyzer, version 3.5; Beijing Kein Research Center for Natural Sciences, **2018**, available from http://sobereva.com/multiwfn.

[25] A. J. W. Thom, E. J. Sundstrom, M. Head-Gordon, *Phys. Chem. Chem. Phys.* **2009**, *11*, 11297.

[26] a) M. v. Hopffgarten, G. Frenking, *WIREs Comput. Mol. Sci.* **2012**, *2*, 43; b) G. Te Velde, F. M. Bickelhaupt, E. J. Baerends, V. Fonseca Guerra, S. J. A. van Gisbergen, J. G. Snijders, T. Ziegler, *J. Comput. Chem.* **2001**, *22*, 931.

[27] For more details about EDA analysis using open-shell fragments, see: https://www.scm.com/doc/ADF/Examples/PCCP_Unr_BondEnergy.html

[28] a) A. D. Becke, K. E. Edgecombe, *J. Chem. Phys.* **1990**, *92*, 5397; b) M. Kohout, A. Savin, *Int. J. Quantum Chem.* **1996**, *60*, 875.

[29] M. Kohout, DGrid, version 5.0, Dresden, **2017**; available from http://www2.cpfs.mpg.de/~kohout/dgrid.html.

[30] Paraview, version 5.2, Kitware Inc., Clifton Park, New York, USA, **2016**; available from http://www.paraview.org.
